# Phytocannabinoids as Novel SGLT2 Modulators for Renal Glucose Reabsorption in Type 2 Diabetes Management

**DOI:** 10.3390/ph18081101

**Published:** 2025-07-24

**Authors:** Raymond Rubianto Tjandrawinata, Dante Saksono Harbuwono, Sidartawan Soegondo, Nurpudji Astuti Taslim, Fahrul Nurkolis

**Affiliations:** 1 Center for Pharmaceutical and Nutraceutical Research and Policy, Faculty of Biotechnology, Atma Jaya Catholic University of Indonesia, Jakarta 12930, Indonesia; 2 Dexa Laboratories of Biomolecular Sciences, Dexa Medica, Industri Selatan V PP-7, Jababeka 2, Cikarang 17550, Indonesia; 3 Division of Endocrinology, Metabolism, and Diabetes, Department of Internal Medicine, Faculty of Medicine Universitas Indonesia, Dr. Cipto Mangunkusumo National Referral Hospital, Jakarta 10430, Indonesia; 4 Metabolic, Cardiovascular, and Aging Cluster, Faculty of Medicine Universitas Indonesia, The Indonesian Medical Education and Research Institute, Jakarta 10430, Indonesia; 5 Diabetes Connection & Care, Eka Hospital BSD, South Tangerang 15321, Indonesia; 6 Division of Clinical Nutrition, Department of Nutrition, Faculty of Medicine, Hasanuddin University, Makassar 90245, Indonesia; 7 Institute for Research and Community Service, State Islamic University of Sunan Kalijaga (UIN Sunan Kalijaga), Yogyakarta 55281, Indonesia; 8 Basic Medical Science, Faculty of Medicine, Universitas Airlangga, Surabaya 60115, Indonesia; 9 Medical Research Center of Indonesia, Surabaya 60286, Indonesia

**Keywords:** phytocannabinoids, SGLT2, type 2 diabetes mellitus, renal glucose reabsorption, in silico docking, cannabidiol, tetrahydrocannabivarin, β-caryophyllene

## Abstract

**Background**: Sodium–glucose cotransporter 2 (SGLT2) inhibitors have transformed type 2 diabetes mellitus (T2DM) management by promoting glucosuria, lowering glycated hemoglobin (HbA1c), blood pressure, and weight; however, their use is limited by genitourinary infections and ketoacidosis. Phytocannabinoids—bioactive compounds from *Cannabis sativa*—exhibit multi-target pharmacology, including interactions with cannabinoid receptors, Peroxisome Proliferator-Activated Receptors (PPARs), Transient Receptor Potential (TRP) channels, and potentially SGLT2. **Objective**: To evaluate the potential of phytocannabinoids as novel modulators of renal glucose reabsorption via SGLT2 and to compare their efficacy, safety, and pharmacological profiles with synthetic SGLT2 inhibitors. **Methods**: We performed a narrative review encompassing the following: (1) the molecular and physiological roles of SGLT2; (2) chemical classification, natural sources, and pharmacokinetics/pharmacodynamics of major phytocannabinoids (Δ^9^-Tetrahydrocannabinol or Δ^9^-THC, Cannabidiol or CBD, Cannabigerol or CBG, Cannabichromene or CBC, Tetrahydrocannabivarin or THCV, and β-caryophyllene); (3) in silico docking and drug-likeness assessments; (4) in vitro assays of receptor binding, TRP channel modulation, and glucose transport; (5) in vivo rodent models evaluating glycemic control, weight change, and organ protection; (6) pilot clinical studies of THCV and case reports of CBD/BCP; (7) comparative analysis with established synthetic inhibitors. **Results**: In silico studies identify high-affinity binding of several phytocannabinoids within the SGLT2 substrate pocket. In vitro, CBG and THCV modulate SGLT2-related pathways indirectly via TRP channels and CB receptors; direct IC_50_ values for SGLT2 remain to be determined. In vivo, THCV and CBD demonstrate glucose-lowering, insulin-sensitizing, weight-reducing, anti-inflammatory, and organ-protective effects. Pilot clinical data (n = 62) show that THCV decreases fasting glucose, enhances β-cell function, and lacks psychoactive side effects. Compared to synthetic inhibitors, phytocannabinoids offer pleiotropic benefits but face challenges of low oral bioavailability, polypharmacology, inter-individual variability, and limited large-scale trials. **Discussion**: While preclinical and early clinical data highlight phytocannabinoids’ potential in SGLT2 modulation and broader metabolic improvement, their translation is impeded by significant challenges. These include low oral bioavailability, inconsistent pharmacokinetic profiles, and the absence of standardized formulations, necessitating advanced delivery system development. Furthermore, the inherent polypharmacology of these compounds, while beneficial, demands comprehensive safety assessments for potential off-target effects and drug interactions. The scarcity of large-scale, well-controlled clinical trials and the need for clear regulatory frameworks remain critical hurdles. Addressing these aspects is paramount to fully realize the therapeutic utility of phytocannabinoids as a comprehensive approach to T2DM management. **Conclusion**: Phytocannabinoids represent promising multi-target agents for T2DM through potential SGLT2 modulation and complementary metabolic effects. Future work should focus on pharmacokinetic optimization, precise quantification of SGLT2 inhibition, and robust clinical trials to establish efficacy and safety profiles relative to synthetic inhibitors.

## 1. Introduction

Phytocannabinoids are the main representatives of a new group of naturally occurring compounds, produced in the plant of Cannabis sp. [1]. Due to the increasing prevalence of diabetes mellitus, in this work, special attention will be devoted to phytocannabinoids that are relevant to glycemic control. Type 2 diabetes mellitus (T2DM) is a metabolic disease characterized by persistent hyperglycemia resulting from the combination of insulin resistance and impaired β-cell function [2,3].

Inhibitors of sodium–glucose cotransporter 2 (SGLT2) of the renal proximal tubules have emerged as effective therapies for treating diabetes. Synthetic SGLT2 inhibitors can lower glycated hemoglobin (HbA1c) and blood pressure as well as reduce diabetic complications [4,5]. However, the adverse effects of these compounds limit their clinical utility in the treatment of diabetes [6]. Phytocannabinoids can be effective modulators of SGLT2 and can be of potential interest in managing renal glucose reabsorption. In silico, in vitro, and in vivo studies suggested the favorable interaction of phytocannabinoids with SGLT2 and their possible involvement in glycemic control [7]. In addition to these effects, some phytocannabinoids such as cannabidiol (CBD), tetrahydrocannabivarin (THCV), and β-caryophyllene exhibited several organ-protective activities that could control the glycemic effects and the associated complications of this metabolic condition [8].

On the other hand, the use of synthetic cannabinoid receptor 1 (CB1R) antagonists such as rimonabant has been prohibited due to their psychiatric side effects [9]. In contrast, phytocannabinoids such as cannabidiol and tetrahydrocannabivarin, produced by cannabis, exhibit antagonistic activity on CB1R signaling and do not show any adverse side effects such as psychoactive effects, depression, or anxiety in early studies [10,11], thereby serving as potential candidates for the treatment of diabetes and its complications, including those responsive to SGLT2 inhibition. In addition to these specific phytocannabinoids, *Cannabis sativa* also produces a substantial number of other phytocannabinoids, alongside terpenes and flavonoids, which contribute to its therapeutic potential against insulin resistance, diabetes, and its complications through synergistic “entourage effects” [12,13], further supporting a multi-mechanistic approach that may complement direct SGLT2 modulation.

The aim of the present work is to clarify whether phytocannabinoids could serve as a potential target for the treatment of type 2 diabetes mellitus through SGLT2 regulation. This study covers the structural and functional properties of SGLT2 in type 2 diabetes, the main classes and characteristics of phytocannabinoids, phytocannabinoids as the SGLT2 inhibitor target, preclinical and clinical studies of phytocannabinoid–SGLT2 interaction, clinical significance and safety of phytocannabinoids for T2DM, limitations and challenges of phytocannabinoids as treatment for T2DM, future research directions, as well as conclusions and perspectives. The novelty of this review lies in its comprehensive synthesis of multifaceted evidence—from in silico predictions to early clinical data—specifically evaluating the potential of phytocannabinoids not merely as glucose-lowering agents but as multi-target SGLT2 modulators with pleiotropic benefits, thereby providing a direct comparative analysis against conventional synthetic SGLT2 inhibitors and outlining critical pathways for their clinical translation.

## 2. Methods

A comprehensive narrative review was conducted to synthesize and critically evaluate the existing literature on phytocannabinoids as potential SGLT2 modulators for renal glucose reabsorption in type 2 diabetes management. The systematic search encompassed major electronic databases, including PubMed, Web of Science, and Scopus, from their inception up to June 2025. The search strategy employed a combination of keywords such as “Phytocannabinoids,” “SGLT2,” “Type 2 diabetes mellitus,” “renal glucose reabsorption,” “in silico docking,” “cannabidiol,” “tetrahydrocannabivarin,” and “β-caryophyllene,” along with their synonyms and related terms.

Original research articles, reviews, and clinical trial reports that investigated the interaction of phytocannabinoids with SGLT2, their effects on renal glucose reabsorption, or their broader metabolic impact relevant to type 2 diabetes were included. Studies focusing on in silico modeling, in vitro assays, in vivo animal models, and human clinical trials/case reports were all considered. Emphasis was placed on studies providing molecular, physiological, pharmacological, and clinical data related to SGLT2 modulation and T2DM management. Commentaries, editorials, conference abstracts without full paper availability, and studies not directly related to phytocannabinoids or SGLT2 in the context of diabetes were excluded. Studies primarily focusing on synthetic cannabinoids (unless used for comparative purposes) or those lacking sufficient detail on methodology or results were also excluded. Non-English language publications were not included due to resource limitations.

Identified studies were screened by title and abstract, followed by a full-text review for relevance and quality. Data extraction focused on key findings related to the following: (1) the molecular and physiological roles of SGLT2; (2) chemical classification, natural sources, and pharmacokinetics/pharmacodynamics of major phytocannabinoids (Δ^9^-Tetrahydrocannabinol or Δ^9^-THC, CBD, Cannabigerol or CBG, Cannabichromene or CBC, THCV, and β-caryophyllene); (3) in silico docking and drug-likeness assessments; (4) in vitro assays of receptor binding, Transient Receptor Potential (TRP) channel modulation, and glucose transport; (5) in vivo rodent models evaluating glycemic control, weight change, and organ protection; (6) pilot clinical studies of THCV and case reports of CBD/BCP; (7) comparative analysis with established synthetic SGLT2 inhibitors.

Studies were critically appraised for their methodology, results, and conclusions, with particular attention paid to the directness of evidence linking phytocannabinoids to SGLT2 modulation. For in silico studies, we reviewed and evaluated the docking scores, binding affinities, and compliance with drug-likeness rules from the literature. In vitro and in vivo studies were assessed for experimental design, statistical significance, and the relevance of their findings to human physiology. Clinical studies and case reports were reviewed for sample size, study design, reported efficacy, and safety profiles. Comparisons between different phytocannabinoids and with synthetic SGLT2 inhibitors were drawn based on their demonstrated efficacy, safety, and pharmacological mechanisms, highlighting both their advantages and limitations.

## 3. Molecular and Physiological Basis of SGLT2

**Figure 1:**
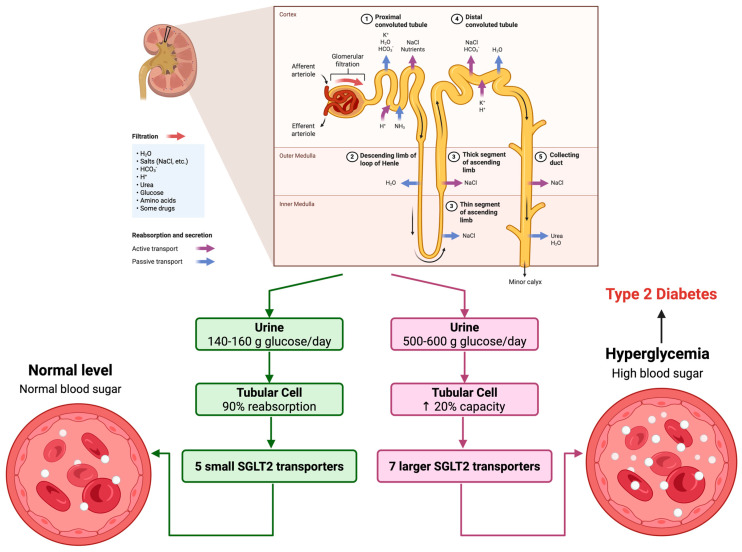
Schematic of SGLT2-mediated glucose reabsorption. Schematic representation of glucose reabsorption in the renal proximal tubule via SGLT2 under normoglycemic (left) and hyperglycemic diabetic (right) conditions, illustrating upregulation of transporter activity in T2DM.

The sodium–glucose cotransporter 2 (SGLT2) is expressed exclusively on the apical membrane of the renal proximal tubule. This is the primary glucose transporter in the kidney responsible for 90% of filtered glucose reuptake from the urine at normal blood glucose levels, with 140–160 g of glucose filtered per day [14]. It has a low affinity but a high capacity to transport glucose into the tubule cells from the filtrate. Its main function is to reduce energy loss, and as such, it is localized to the S1 and S2 segments of the proximal tubule where the glucose concentration in the filtrate is the highest (Figure 1).

SGLT2 is sensitive to physiological inputs such as glycemic state, insulin, and inflammation. This suggests that SGLT2 expression and activity can be enhanced by elevated glucose levels or pathological circumstances [14]. Genetic studies indicate that patients with familial renal glucosuria and SGLT2 knockout mice show increased glycosuria with no significant changes in electrolyte homeostasis [15,16]. This further confirms its specificity and demonstrates its pharmacological usefulness.

In T2DM, hyperglycemia increases glucose filtered through the kidneys, causing an upregulation of SGLT2 protein levels and increased activity of the transporter [17]. This compensatory effect exacerbates hyperglycemia by increasing total tubular reabsorption capacity of glucose by 20% in T2DM, with an estimated maximum glucose reabsorption of 500–600 g per day [14]. Increased glucose reabsorption also contributes to nephron damage from the elevated flux of glucose throughout this organ. Chronic hyperglycemia and the consequent increase in filtered glucose load are not only common in T2DM but also in type 1 diabetes mellitus, which promotes overactivity of this transporter [18].

Pharmacological or genetic deletion of SGLT2 reduces the renal threshold for glucose and leads to marked glycosuria in both mice and humans, which SGLT1 cannot compensate for [14]. From a structural perspective, SGLT2 is a transmembrane protein. While a detailed molecular structure is complex, in silico studies targeting SGLT2 have specifically utilized its crystal structures, such as the 3BAJ crystal structure, to identify potential binding sites for modulators [19]. These computational analyses reveal that phytocannabinoids exhibit high binding affinities within the SGLT2 substrate-binding pocket, engaging in key interactions like hydrophobic and hydrogen bonding, which mimic the mechanisms of action observed with existing synthetic inhibitors. This characteristic binding within the substrate pocket is fundamental to its role in glucose transport and its interaction with inhibitory molecules.

Studies on humans with T2DM reveal that inhibition of SGLT2 reduces HbA1c by 0.5–0.7% in 52 weeks, regardless of the population [20]. The self-limiting action that is controlled by plasma glucose levels minimizes the risk of hypoglycemia by acting independently of insulin action. SGLT2 inhibitors also decrease postprandial glucose and glycemic variability [21]. SGLT2 inhibition causes osmotic diuresis that reduces systolic blood pressure by 2–6 mmHg, and there is an observed weight loss of 2–4 kg [14,22]. Studies report that it decreases hyperfiltration and the glomerular filtration rate, reducing the progression of diabetic nephropathy [22]. Albuminuria is diminished while vascular function in the kidneys is enhanced upon SGLT2 inhibition in rodents and in patients with T2DM [22]. By lowering inflammatory biomarker levels, SGLT2 inhibitors display renoprotective effects that are critical for treatment and for alleviating kidney damage.

Synthetic SGLT2 inhibitors display IC_50_ values for SGLT2 and SGLT1 that range from 0.46 nM to 2334 nM for human SGLT2 [23]. This difference depends on the potency and selectivity of inhibitor drugs, with GCC5694A and Compound 2 showing IC_50_ values of 0.46 nM and 0.64 nM, respectively, which are much lower than, for example, dapagliflozin [23]. This high selectivity for SGLT2 is extremely beneficial in reducing GI side effects caused by the potent inhibition of SGLT1. However, SGLT1 inhibition is not completely avoided by SGLT2 inhibitors. For example, empagliflozin inhibits SGLT2 activity with a significantly higher potency and less potency in SGLT1, which accounts for reduced, but not eliminated, side effects. Inhibitors also range in pharmacokinetic attributes such as bioavailability, which in synthetic inhibitors can exceed 78% [23].

Optimization of oral absorption is paramount in novel treatment drugs. Phytocannabinoids, such as cannabidiol, inherently suffer from low oral bioavailability, often less than 10%, primarily due to their poor water solubility, lipophilicity, and extensive first-pass hepatic metabolism. To circumvent these pharmacokinetic limitations and enhance systemic exposure, various advanced pharmaceutical formulation technologies are being explored. These include the development of self-emulsifying drug delivery systems (SEDDS), modified crystal systems, solid-state formulations, nanoemulsions, liposomes, and other lipid-based formulations [24,25]. The primary conditions and aims of these optimization methods are to improve aqueous solubility, enhance lymphatic absorption, decrease hepatic exposure, promote overall gastrointestinal absorption, and ensure drug stability for sustained release. Ultimately, these efforts seek to reduce inter-individual variability in systemic exposure, achieve consistent therapeutic levels, and provide a predictable and reliable therapeutic response in patients. While promising, many of these delivery strategies are still in preclinical or early clinical phases, facing challenges in large-scale manufacturing and widespread clinical validation.

Synthetic inhibitors cause an elevated risk of genital and urinary tract infections, which is hypothesized to be from increased glycosuria altering osmolality and increasing the abundance of bacteria in urine. In the long term, SGLT2 inhibitors display side effects of euglycemic diabetic ketoacidosis [22]. This and other side effects, such as dehydration or electrolyte imbalances, are limited with synthetic inhibitors, but still require continuous monitoring due to long-term complications from sustained glycosuria [14]. To reduce the onset of side effects, phytocannabinoids with antimicrobial and anti-inflammatory activity are explored as possible modulators of SGLT2. Additionally, phytocannabinoids are observed to display tissue-protective and antioxidative properties that may minimize damage in both kidney and pancreatic tissues [23].

SGLT2 has paved the way for synthetic SGLT2 inhibitors, but as detailed above, they have many limitations. The multiple pharmacological properties of phytocannabinoids provide an unexplored area that may limit the occurrence of serious side effects experienced with synthetic inhibitors while retaining, or even exceeding, SGLT2-inhibiting action.

## 4. Phytocannabinoids: Classification and Pharmacology

The heterogeneous chemical nature and pharmacological properties of phytocannabinoids extracted from *C. sativa* create a strong basis for their potential uses in metabolic health. From a diabetic point of view, this makes a further exploration into their classification, natural sources, pharmacokinetics, and mechanism of action mandatory for a broad understanding of their SGLT2-inhibiting capabilities.

### 4.1. Major Classes of Phytocannabinoids

**Figure 2:**
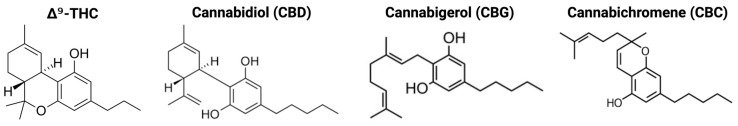
Chemical structures of major phytocannabinoids. Structures of the four principal phytocannabinoids evaluated for SGLT2 modulation: Δ^9^-THC, cannabidiol (CBD), cannabigerol (CBG), and cannabichromene (CBC).

Phytocannabinoids derived from *C. sativa* have diverse structures, which provide them with multiple physiological functions (Figure 2). The structural variation of 120 naturally occurring cannabinoids, categorized into 11 chemical subclasses, leads to various pharmacological properties, including a potential role in SGLT2 modulation [26]. Thus, each individual phytocannabinoid may target SGLT2 with unique potential in renal glucose reabsorption in T2DM.

The main phytocannabinoids, Δ9-THC, CBD, CBG, and CBC, differ considerably in pharmacological activity because of differences in their receptor binding affinity, mode of action at various receptors, and also through cannabinoid and non-cannabinoid signaling pathways [27]. For example, CBG has moderate binding affinity at CB1 and CB2 receptors and also behaves as a potent antagonist at the TRPM8 channel as well as an activator at TRPA1 and TRPV1 channels, which suggests that CBG can activate or inhibit several molecular targets [28,29]. Additional studies are necessary to clarify these specific mechanisms so that SGLT2 modulation can be specifically enhanced by each phytocannabinoid and off-target adverse effects can be minimized.

Psychoactive and non-psychoactive phytocannabinoids need to be considered to evaluate their efficacy in metabolic health, with more attention to the use of non-psychoactive compounds, due to the central psychoactive side effects of Δ9-THC. Unlike Δ9-THC, non-psychoactive phytocannabinoids CBD and THCV act as CB1 antagonists in the periphery without central psychoactive effects [30]. THCV increases glucose tolerance, insulin sensitivity, and pancreatic β-cell function in animal models and human volunteers in clinical trials [31]. These results encourage efforts to better understand non-psychoactive phytocannabinoids to minimize psychoactive effects and provide safety for chronic diseases, such as diabetes, while exploring their direct or indirect SGLT2-modulating capabilities.

Phytocannabinoids may have synergistic effects through “entourage effects.” The “entourage effects” of phytocannabinoids refer to the synergistic benefits of multiple constituents found in *C. sativa*, that is, cannabinoids, terpenes, and other compounds [26]. Evidence suggested that whole-plant extracts of cannabinoids are more efficacious compared with any single phytocannabinoid [32]. This complex interplay of compounds may offer a broader spectrum of anti-diabetic activity, potentially complementing SGLT2 modulation by addressing multiple pathological pathways simultaneously. The specific role of each subclass still needs to be identified. Thus, systematic evaluations and multimodal therapies with subclasses of cannabinoids are very necessary for improved glycemic control in T2DM patients.

Δ9-THC, CBD, CBG, and CBC in *C. sativa* are first produced as the carboxylated acids, which are then decarboxylated by heat or aging to their neutral metabolites [27]. The use of multi-cannabinoid extracts can cause synergistic “entourage effects” with cannabinoids, terpenes, and flavonoids of cannabis [31]. These “entourage effects” can be beneficial in chronic diseases, such as T2DM [26]. The multi-target approach in pharmacological treatment for complex diseases is a trend; however, the interactions among various active chemicals make the process complex and can lead to adverse effects. Oral bioavailability of CBD and CBG is low due to their highly lipophilic and non-polar character, very low aqueous solubility, and pre-systemic hepatic clearance [33].

CBG has moderate binding affinity at both CB1 and CB2 receptors and acts as a potent antagonist at the TRPM8 channel, and also as an activator at TRPA1 and TRPV1 channels, indicating that one single phytocannabinoid can activate or inhibit many kinds of molecular targets involved in glucose balance and the renal system [27]. These effects can target multiple pathogenic factors of T2DM. However, such a polypharmacological profile increases the risk of off-target actions. This can be avoided by further structural modification. CB1 and CB2, PPARs, and TRP channels are not all the targets of phytocannabinoids. By acting on these receptors and TRP channels, phytocannabinoids, specifically non-psychoactive ones, are able to reduce hyperglycemia and act as anti-inflammatory and renoprotective molecules, with potential use in controlling T2DM [30]. CBD and THCV exhibit antagonist or inverse agonist activity in the CB1 receptors and, in that way, they can block CB1-mediated responses that may cause psychoactive effects [34]. This makes them suitable for prolonged pharmacological use. CB2 receptors are the primary target for β-caryophyllene (BCP) and its cannabinoid activity. BCP is a CB2 full agonist and a PPAR activator, which can cause anti-inflammatory and antioxidative properties [8]. BCP can cause protective benefits against oxidative stress in the pancreas and kidneys of metabolic syndrome (MS) mice, which have insulin resistance [35]. Thus, these observations support the therapeutic potential of BCP, as it affects diabetes-related disease pathologies.

The potential of phytocannabinoids that can affect the SGLT2 receptor along with many other targets and that have the capacity to increase insulin and decrease inflammation makes them strong candidates for T2DM treatment. The challenges posed by the poly-target nature and the uncertain dose–response relationship need to be addressed in more depth. In addition, the pharmacokinetic differences and the inter-individual diversity of patients may pose some clinical issues that should be taken into consideration [8,30]. These agents could be useful for metabolic management.

### 4.2. Natural Sources of Phytocannabinoids

**Figure 3:**
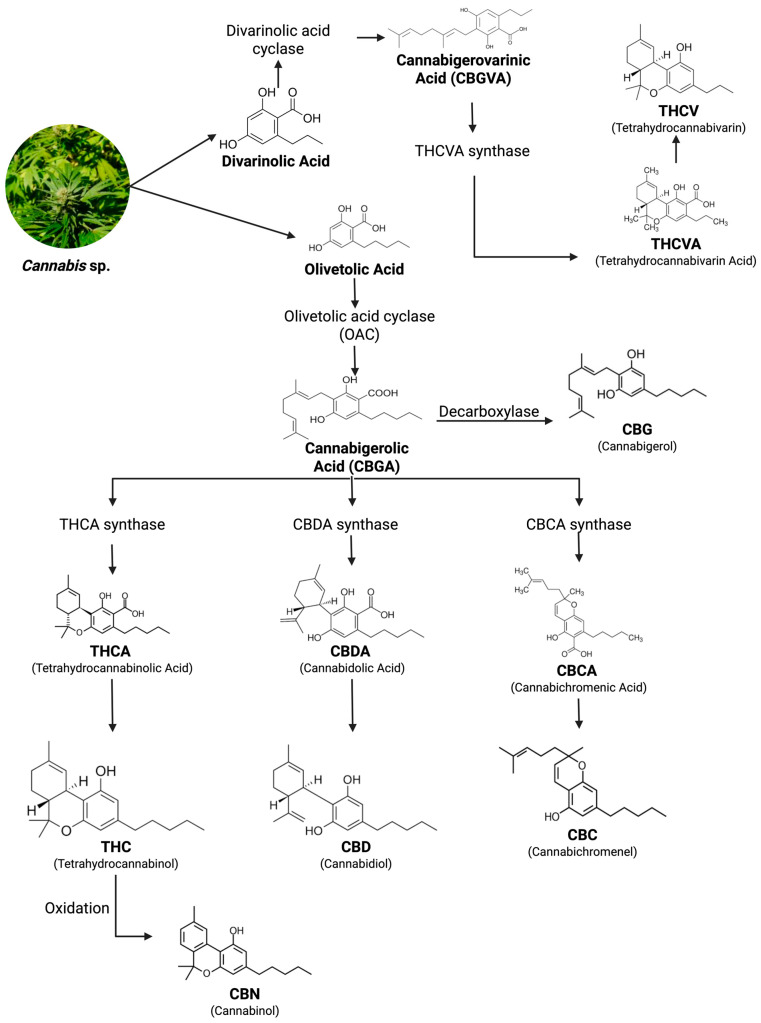
Localization of phytocannabinoid biosynthesis in cannabis trichomes.

Phytocannabinoids are mainly obtained from the *C. sativa* plant, characterized by over 120 cannabinoids found, and classified into 11 chemical subclasses based on structural features (Figure 3). Such features are mostly determined by the arrangement of their functional groups, which leads to different bioactivities. From these subclasses, Δ9-THC, CBD, CBG, and CBC stand out because of their chemical diversity, which suggests a high probability of finding natural compounds able to modulate molecular targets related to diabetes [26].

From all cannabinoids, minor cannabinoids have received little attention due to their low presence [36,37]. On the other hand, as minor cannabinoids are mostly present in lower concentrations compared to major cannabinoids, their selective activity on metabolic pathways is gaining relevance. THCV could modulate glucose homeostasis and insulin sensitivity, which makes this cannabinoid a suitable candidate for SGLT2 modulation [38]. The exploration of the composition profile of C. sativa is growing thanks to the analytical and chemical techniques available, which allow a proper identification and quantification of low-abundance compounds. Furthermore, profiling techniques guarantee the reproducibility of the studies when the composition of cannabinoids is a key feature for assessing results. Reproducibility is a valuable element in determining candidate cannabinoids for SGLT2 modulation [27]. This reproducibility is critical for the consistent development and reliable evaluation of phytocannabinoids as SGLT2-targeting therapeutics.

The concentration of individual cannabinoids varies between different parts of the plant, as it can range from 20% to less than 1%. Unfertilized female flowers contain the highest concentration of cannabinoids in their trichomes (Figure 3) [27]. The cannabinoid content in the trichome is higher than in any other plant organ [39]; therefore, they constitute the main source of cannabinoid isolates. For industrial cannabinoid isolation, trichomes are frequently collected to obtain higher yields. The extraction of all cannabinoids may require the harvest of several plant organs, such as flowers, leaves, stems, and roots. The problem with this procedure is that the plant matrix interferes with the extraction process; therefore, the isolation of cannabinoid extracts with pharmaceutical quality standards is hard to achieve, making this process expensive and challenging. Thus, most pharmaceutical formulations based on *C. sativa* are standardized cannabinoid extracts exclusively obtained from unfertilized female flowers [27].

Cannabinoid content in cannabis depends mostly on its genes, the conditions in which it grows, and post-harvest processing. In other words, it depends on environmental and agronomic factors. As far as these last factors are concerned, the variability in the composition profile of cannabinoids is caused by differences in soil composition, plant lighting, nutrient supply, and harvest time, among others [27]. To ensure the reproducibility of experiments performed with cannabis, the plants’ cultivation, harvest time, and storage conditions must be highly controlled. The composition variability in cannabinoids must be ensured to the best extent possible in the initial experimental phases. Nowadays, to assess this variability, high-resolution chromatographic fingerprinting of different cannabis samples allows for to comparison of their chemical profile and to predict the pharmacological effects of phytocannabinoids [27].

Phytocannabinoids represent an outstanding potential for the treatment and management of diabetes for multiple reasons. Yet, there are still key concerns to address in order to guarantee reproducible outcomes for the research and pharmacological application of this chemical subclass.

### 4.3. Pharmacokinetic and Pharmacodynamic Profiles

The pharmacokinetics and pharmacodynamics of phytocannabinoids significantly influence their potential to modulate sodium–glucose cotransporter 2 (SGLT2) for T2DM management. Cannabidiol (CBD), for instance, suffers from serious pharmacokinetic issues that limit its therapeutic utility. Its very low bioavailability when delivered orally, less than 10%, is due to its poor water solubility, lipophilicity, and extensive first-pass hepatic metabolism, which results in reduced gastrointestinal absorption [33]. Varying levels of dietary fat content and metabolic enzyme expression across individuals add to the inconsistent absorption levels of CBD, further contributing to the ambiguity of its bioavailability. Several new delivery methods are currently under development, including self-emulsifying drug delivery systems (SEDDSs) and modified crystal systems that improve aqueous solubility and lymphatic absorption as well as decrease hepatic exposure [40,41]. While these developments show great potential, their implementation faces scaling and standardization problems. Optimizing these drug delivery systems is crucial for CBD to achieve the dose titration necessary to maximize therapeutic benefit and avoid subtherapeutic exposures, thereby increasing the utility of CBD as an SGLT2 modulator.

The variable receptor binding and multiple biological targets of other phytocannabinoids, such as cannabigerol (CBG), increase the pharmacodynamic complexity of this group of compounds. CBG binds both CB1 and CB2 receptors with moderate affinities (CB1: Ki = 381–897 nM; CB2: Ki = 2.6 μM/153 nM, respectively), allowing for CB receptor activation in the kidneys, liver, and pancreas without the psychoactive consequences observed from other higher-affinity CB1 agonists [27]. Aside from its affinity to CB1 and CB2 receptors, CBG also activates TRPA1 and TRPV1 channels and antagonizes the TRPM8 channel (IC_50_ = 0.16 μM), suggesting that its modulatory activity on SGLT2 may occur through the influence of ion channels that are involved in tubular activity in the kidney [27]. In addition to that, studies on the structure–activity relationship of phytocannabinoids have shown that modifications made in the phytocannabinoid structure have drastically altered their preference for different cannabinoid receptors and/or other targets [42], indicating that phytocannabinoids would need to be extensively engineered at the core in order to avoid binding to unwanted receptors. The polypharmacological mechanism of phytocannabinoids allows for pleiotropic anti-inflammatory and metabolic effects, which is beneficial; however, the activation of many receptors and/or channels increases the possibility of adverse effects and drug interactions. It is important to understand the exact target receptors that induce the desired cellular mechanisms, thereby preventing unwanted actions in other tissues and increasing drug safety.

Phytocannabinoids such as β-caryophyllene (BCP), have a favorable oral bioavailability, a non-psychoactive effect, and may have the potential to modulate the activity of SGLT2 and thus be a useful drug in patients with T2DM [43]. BCP activates CB2 receptors, which are the predominant CB receptor responsible for the immunomodulatory and anti-inflammatory actions of BCP, without CB1 receptor activity and thus its psychoactive effects [8]. BCP also acts as a PPAR-α/γ agonist [44], two nuclear receptors responsible for the regulation of fatty acids, energy storage, and glucose metabolism. These pharmacodynamic activities of BCP may reduce several of the symptoms and complications of T2DM and make it an attractive alternative or supplement to synthetic small molecules. In addition, BCP displays antagonistic activity at nicotinic acetylcholine receptors, activates various TRP channels, and inhibits the TLR4-mediated inflammatory pathways, therefore targeting several systems in the body [8]. Furthermore, BCP has a highly lipophilic nature, allowing it to readily penetrate biological barriers. While these advantages have created much interest in the drug, the therapeutic potential of BCP as an alternative treatment strategy for T2DM needs to be further evaluated in controlled clinical studies, as does the efficacy and toxicity data of prolonged BCP treatment.

Poor systemic exposure to phytocannabinoids has been an ongoing challenge for drug delivery development. Their bioavailability is greatly impacted by hepatic metabolism due to the high variability of hepatic CYP450 expression [33]. This variation results in broad inter-individual differences in systemic exposure and efficacy following oral administration, as well as the risk of drug–drug interaction when combined with other pharmaceutical molecules. Additionally, the lipophilicity of cannabinoid-based drugs can compromise their bioavailability by altering the bloodstream concentration and the subsequent therapeutic action [45]. For this reason, solubility problems and bioavailability remain two of the most pressing challenges with respect to phytocannabinoid-based drug development. Therefore, in addition to focusing on strategies to circumvent rapid hepatic metabolism of cannabinoids by the creation of nanoemulsions, liposomes, lipid-based formulations (e.g., SEDDS), solid-state crystallization systems, and amorphous solids to promote absorption and increase the amount of drug in the circulation, future clinical trials should aim at large-scale randomized controlled trials to ensure that formulations are effective at a broader clinical level and scalable from a regulatory perspective.

CBG is able to modulate several TRP channels (TRPA1, TRPV1, and TRPM8), which may offer the opportunity for a broader range of activities or mechanisms of action [27]. These TRP channels are transmembrane proteins that respond to a variety of stimuli and allow for cellular excitability, calcium influx, and secretion of insulin in the pancreas. Many cannabinoids exert both organ-protective activities (neuroprotection, vasoprotection, cardioprotection, etc.) and metabolic regulatory functions, which is ideal for the prevention of diabetic complications that result from altered carbohydrates and fat metabolism [8]. However, similar to phytocannabinoids with activity on several receptors, CBG’s effects on multiple TRP channels could potentially generate off-target actions in the brain, renal tubules, and pancreatic islet cells. The main challenge will be to target one channel without unintentionally interacting with other ones at the targeted cells in vivo.

Overall, while phytocannabinoids have several inherent challenges regarding drug delivery and off-target effects, it will be important to assess these hurdles and devise approaches that will allow the use of phytocannabinoids in SGLT2-based drugs for T2DM.

### 4.4. Mechanisms of Action

**Figure 4:**
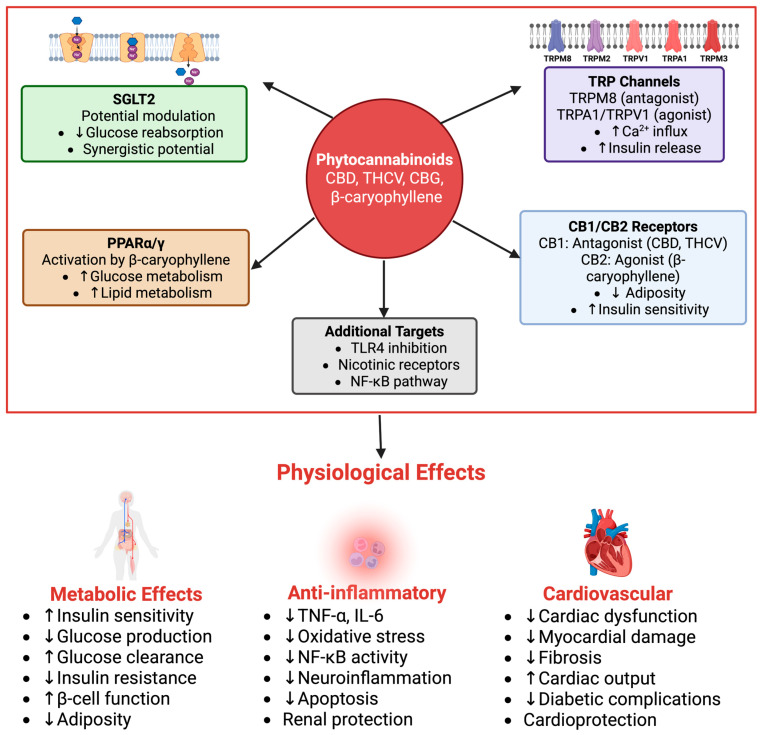
Phytocannabinoid–target interaction network. Diagrammatic network of phytocannabinoid interactions with molecular targets relevant to T2DM: SGLT2, CB_1_/CB_2_ receptors, TRP channels (TRPM8, TRPA1, TRPV1), and PPARα/γ/γ. ↑: Increase/Rise; ↓: Decrease/Drop.

Phytocannabinoids have been found to modulate glucose homeostasis and inflammation through multiple molecular targets, with relevance to SGLT2 modulation (Figure 4). Acting through CB1 and CB2 cannabinoid receptors, PPARs, and TRP channels, these compounds offer multifaceted effects relevant to antihyperglycemic and anti-inflammatory outcomes that can synergize with or complement SGLT2 inhibition. CB2 activation by, for instance, β-caryophyllene, was reported to reduce TNF-α and IL-6 levels, which are thought to be a pivotal part of diabetic nephropathy and insulin resistance [8,46]. Although this effect is beneficial to local renal tissues, further research is needed to see whether the decrease in TNF-α and IL-6 reduces insulin resistance. Variations in bioavailability and receptor activation by different phytocannabinoids require further studies to determine the broader systemic effects on glucose regulation.

A direct interaction with SGLT2 has been primarily suggested by in silico evidence, where several phytocannabinoids like cannabichromene, cannabicyclol, and delta(9)-tetrahydrocannabinolic acid show high binding affinities within the SGLT2 substrate-binding pocket [47], mimicking the mechanism of action of synthetic inhibitors. This indicates a potential for phytocannabinoids to directly modulate SGLT2 activity, although direct in vitro functional assays demonstrating specific SGLT2 inhibition potency (e.g., IC_50_ values) are still largely underexplored for many of these compounds.

Beyond potential direct SGLT2 interaction, specific phytocannabinoids exert various indirect effects. For instance, β-Caryophyllene also activates PPAR-α and PPAR-γ, regulating glucose and lipid metabolism in vitro, resulting in enhanced insulin sensitivity [8]. While these actions are not direct SGLT2 modulations, improved insulin sensitivity and reduced systemic inflammation can indirectly alleviate the metabolic burden on the kidneys, potentially mitigating SGLT2 upregulation in hyperglycemic states and complementing the benefits of SGLT2 inhibition. Furthermore, CB2 signaling contributes to increased calcium influx into pancreatic β-cells to release more insulin and decrease blood glucose levels, an effect that could synergize with SGLT2 inhibition for more efficient glucose control. PPAR activation also contributes to glucose control, yet more studies are required to investigate the exact influence of this interaction and its combination with SGLT2 activity. Finally, the possibility of off-target effects requires an in vivo investigation to further expand the structure–activity relationship and determine pharmacological specificity.

Phytocannabinoids such as cannabigerol (CBG) have multiple interactions with the TRP channels (TRPM8, TRPA1, and TRPV1), which also play an important role in diabetes pathophysiology and renal glucose handling [27]. CBG acts as an inhibitor of TRPM8, influencing Ca2+ handling in pancreatic β-cells and potentially stimulating direct metabolic control of glucose levels by increasing insulin production, while TRPA1 and TRPV1 are activated, reducing neurogenic inflammation and increasing insulin sensitivity [48]. In general, this also shows that phytocannabinoids have non-cannabinoid receptor interactions and therefore could be relevant for regulating diabetes through multiple mechanisms, with benefits in SGLT2 regulation. However, similar to CB2 receptor agonism and PPAR activation, TRPM8, TRPA1, and TRPV1 have multiple interactions, which also lead to a complex and not yet fully understood set of effects. Moreover, each compound may have its own preferences regarding the subtypes that are activated, which adds to the complexity of phytocannabinoid treatments and points out the necessity of establishing a specific dose–response relationship for each compound.

Since phytocannabinoids target multiple pathways, combined treatments may result in benefits to glucose management, renal glucose reabsorption, and systemic inflammation, which could lead to synergistic antihyperglycemic benefits of SGLT2 inhibition. Moreover, the ability of phytocannabinoids to compensate for compensatory mechanisms such as glycemic escape during synthetic SGLT2 inhibitor treatments represents a rational foundation for developing combinatorial therapies. Still, little is known about how the strategic effects are integrated with SGLT2 and if phytocannabinoids can mimic the efficacy of synthetic inhibitors on SGLT2 with their known health benefits.

Since CB2 receptor activation reduced inflammatory cytokines, reactive oxygen and nitrogen species, as well as apoptosis, CB2 receptors play a vital part in improving and protecting renal function in diabetic rats and in providing beneficial metabolic and cardiovascular health [8,46]. Studies have found that the CB2-selective agonist β-caryophyllene inhibits NF-κB production and reduces oxidative stress [8]. This supports the possibility that CB2 receptor activation is beneficial to renal function in cases of diabetes and thus works as a synergistic pathway to regulate renal health. In addition, CB2 signaling contributes to increased calcium influx into pancreatic β-cells to release more insulin and, as a result, decrease blood glucose levels [49]. This shows that this novel CB2-stimulating pathway could result in overall glucose homeostasis and be important in treating diabetic nephropathy. This effect could be used together with SGLT2 inhibition to regulate blood glucose control more efficiently in diabetic patients, but further research is required to confirm and support these initial studies.

Phytocannabinoids are also responsible for modulating TRP channels and regulating diabetes. CBG, as an antagonist of TRPM8, increases Ca2+ uptake in pancreatic β-cells, releasing insulin. CBG also activates TRPA1 and TRPV1 to lower glucose production by inhibiting glucagon secretion [50]. Furthermore, TRPA1 activation has also shown decreased insulin resistance and improved glycemic control [27]. Lastly, these TRP channel interactions of phytocannabinoids also have anti-inflammatory effects that further improve symptoms. These preliminary findings suggest that phytocannabinoids can alleviate diabetic nephropathy and improve overall glucose management; however, more data are required to determine effective phytocannabinoid treatments.

CBD and THCV were found to act as CB1 antagonists, and both do not have psychoactive effects, which prevents the known effects seen from rimonabant. CBD has been shown to reduce adiposity and improve insulin sensitivity. Also, CB1 is expressed in the periphery more so than the central nervous system and therefore may have more metabolic effects in the peripheral system [51]. These findings imply the possibility of treating T2DM in humans, as it has similar metabolic profiles but may be avoided by neuropsychiatric complications [30]. Therefore, additional studies are warranted to investigate the effects in diabetic rodent models and in vitro settings to test whether they can improve blood glucose control compared to rimonabant, with less risk.

The effects of β-caryophyllene act as a full CB2 agonist, activate PPAR-α and PPAR-γ, antagonize nicotinic acetylcholine receptors, and inhibit toll-like receptor 4 (TLR4) signaling [8]. β-Caryophyllene is able to lower inflammation and increase metabolic function due to CB2 receptors. β-Caryophyllene lowers glucose production and insulin resistance by activating PPARs in diabetes treatment [52]. β-Caryophyllene is also known to relieve neuroinflammation by blocking nicotinic acetylcholine receptors to reduce chronic brain inflammation and prevent other harmful symptoms associated with multiple sclerosis (MS) [53]. In addition, β-caryophyllene reduces NF-κB activity by antagonizing nicotinic acetylcholine receptors and inhibits pro-inflammatory cytokines in order to decrease oxidative stress [8]. Therefore, with the ability to be involved with multiple signaling pathways, this could result in anti-inflammatory and anti-diabetic activity, ultimately helping with diabetes complications. However, with such potential comes a requirement to test its potential side effects and influence on diabetic patients, and further research must be performed.

Improved calcium influx into pancreatic β-cells, leading to more insulin release, oxidative damage regulation, and regulation of inflammation in the pancreas and the kidneys in diabetic mice under hyperglycemic conditions, are also some of the effects resulting from CB2 activation with phytocannabinoids [54]. By restoring intracellular calcium levels, CB2 receptor stimulation could help preserve the survival of β-cells, whose life cycle has been found to be shortened in diabetics. Moreover, it could also slow the progression of diabetic nephropathy by reducing inflammation and, eventually, reducing the damage and loss of nephrons. This demonstrates the synergistic potential between SGLT2 and CB2 mechanisms in restoring overall glucose balance.

This multi-modal action could complement SGLT2 modulation by addressing the complex pathophysiology of diabetes beyond direct glucose reabsorption inhibition, for example, by reducing systemic inflammation or improving insulin sensitivity. Although complex mixtures of cannabis metabolites seem promising for the treatment of diabetes, the effects of the specific combination of cannabinoids on each of the relevant pathways remain to be explored. The compositional variability of all cannabis extracts makes them unsuitable for standardization. Nevertheless, they may be tailored for personalized treatment regimens to meet individual metabolic demands

## 5. Phytocannabinoid–SGLT2 Interactions

Exploring the molecular interactions between phytocannabinoids and SGLT2 offers hope for new diabetes therapies. From the perspective of in silico, in vitro, and in vivo studies, this section showcases the plant’s ability to affect renal glucose reabsorption. Within the framework of utilizing phytocannabinoids as therapeutics, this section paves the way for new drug development strategies targeting metabolic pathways for diabetes management.

### 5.1. In Silico Evidence

**Figure 5:**
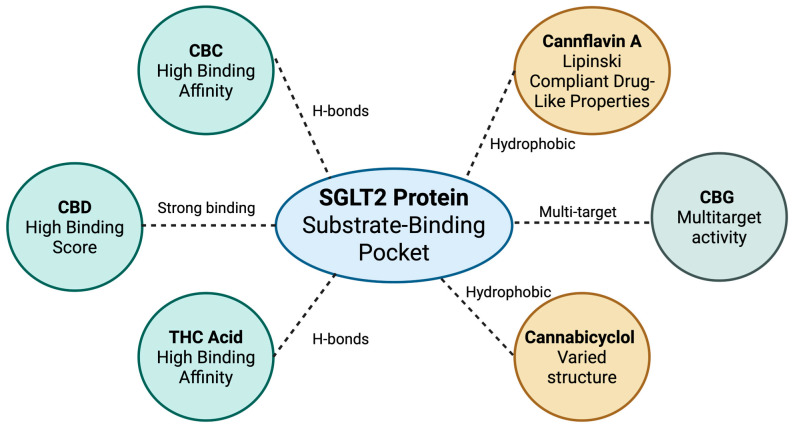
In silico docking poses of top phytocannabinoids in SGLT2.

In silico virtual screening is increasingly valuable for identifying novel modulators of sodium–glucose cotransporter 2 (SGLT2), particularly in the context of phytochemicals from *C. sativa* (Figure 5). Several compounds, like cannabichromene, cannabicyclol, delta(9)-tetrahydrocannabinolic acid, and cannflavin A, have shown high binding affinities and favorable drug-likeness [19]. With SGLT2, especially its 3BAJ crystal structure, as the primary target, virtual screening has demonstrated that these phytochemicals have high binding affinities and form hydrophobic and hydrogen bonds within the substrate-binding pocket, mimicking the mechanism of action of available synthetic inhibitors [19]. This initial in silico evidence represents a crucial and original step, demonstrating that phytocannabinoids, despite their distinct chemical structures from established synthetic SGLT2 inhibitors, possess the molecular prerequisites for direct interaction with this key glucose transporter. This suggests that *C. sativa* possesses the potential to yield SGLT2 modulators that can be developed further through structure-based drug design and optimization.

Drug-likeness studies have focused on physicochemical parameters, evaluating potential phytocannabinoids against Lipinski’s rule of five and the Muegge criteria. Several cannabinoids, including cannabichromene, cannabicyclol, Cannabisin B, Cannabisin C, and cannflavin A, met the required criteria [19], suggesting that they may have optimal physicochemical properties for being orally bioavailable SGLT2 modulators. This computational analysis of affinity against human pancreatic alpha-amylase, paired with the Lipinski’s rule of five and the Muegge criteria, validates these compounds as theoretically plausible drug candidates and can also streamline the drug discovery process by excluding drug candidates with undesirable pharmacokinetic properties early in the process. As such, future efforts can focus on drug candidates with a better likelihood of success, thus enhancing translational relevance. Employing the two criteria will also help decrease the risk of identifying compounds with promising binding energies that lack other important properties, such as solubility or metabolic stability.

Computational docking suggests binding motifs of the phytochemicals against the SGLT2 binding site for structural refinement. Identified phytocannabinoids such as delta(9)-tetrahydrocannabinolic acid and cannabicyclol can potentially broaden the selection pool of candidates to be tested, as they have both high binding affinities for SGLT2 [19] and varied chemical structures. These candidates can then be used in a structure–activity relationship (SAR) study, as they may have interesting functional groups that may improve their efficacy or reduce side effects. This comprehensive approach, with two sets of criteria based on affinity and physicochemical properties, helps select the most promising compounds in silico. In the present case, cannabichromene and cannflavin A meet these two criteria, suggesting they have a greater likelihood to succeed in preclinical and clinical studies [19]. As such, these methods improve resource efficiency and streamline further efforts in in vitro, in vivo, and clinical trials. These methodologies can also be expanded into future studies of other SGLT2 inhibitors or even for the identification of modulators of other renal transporters involved in the development and progression of diabetes.

One of the advantages of using in silico approaches in phytomedicine is the ability to identify compounds with dual activities. This can be achieved through identifying phytochemicals that not only inhibit SGLT2 but also have other beneficial properties for diabetes, such as improved insulin signaling and glycemic regulation, reduction in the inflammation and oxidative stress associated with diabetic nephropathy [55], and even reduction in hepatic gluconeogenesis and fat absorption in obesity. It can also select molecules that may have the most potential based on knowledge gained of the ethnobotanical source, which in this case is *C. sativa*, which can be used for diabetes to not only inhibit SGLT2, but also for treating diabetic complications. For example, cannabigerol (CBG) is known to have moderate affinity for other biological targets, such as cannabinoid receptors, and it can also modulate transient receptor potential (TRP) channels [27], which is not uncommon among phytochemicals. If the compound being considered interacts with multiple targets, computational tools can be used to simulate competition or synergism with other compounds known to interact with one or more of those additional biological targets. Such simulations can optimize the efficacy or safety profiles of such drug candidates by modeling the interaction or competition of multiple chemical entities with several targets in the setting of a metabolic environment. This is especially true for phytochemicals, as they are known to have several biological targets. For example, CBG can regulate renal glucose homeostasis through SGLT2 modulation and modulation of TRP channels [27]. To study the effects of such compound interactions, computational simulation can be used to explore drug–drug and drug–target interactions for the prediction of drug interactions, dose optimization, and toxicity profiles.

In summary, the use of phytocannabinoids as a lead chemical structure for SGLT2 modulation provides a new perspective and direction for current and future therapies and therapeutics of diabetes. In silico, virtual screening and simulations have facilitated the identification of compounds of interest, allowing the rationalization and optimization of therapeutic targets. This can be paired with laboratory experiments to further refine the therapeutic efficacy, as well as ensure a high efficacy, a high safety profile, and a well-defined therapeutic benefit in patients with diabetes.

### 5.2. In Vitro Evidence

In vitro studies have investigated the mechanisms by which phytocannabinoids may influence key molecular targets for glucose regulation in the body, like SGLT2. In addition to moderate binding affinities to CB1 and CB2 receptors reported in cell-based assays for cannabigerol (CBG), an activation of these cannabinoid receptors may indirectly lead to functional outcomes in glucose reabsorption [56,57]. Specifically, CBG displayed Ki values of 381 nM and 153 nM to 2.6 μM for CB1 and CB2, respectively [56]. CB1/2 activation would initiate cellular signal transduction via phosphorylation events of various downstream proteins, likely in the renal proximal tubule and/or islet β-cells due to co-localized cannabinoid receptor expression in these tissues. However, the evidence cited [27] merely documents the cannabinoid receptor binding affinity and signal transduction within kidney and brain cell lines, and thus the impact CBG may have on SGLT2 has yet to be determined.

Furthermore, CBG is a potent TRPM8 antagonist with an IC_50_ of 0.16 μM [27]. Additionally, it acts as a TRPA1 agonist with an EC50 value of 3.4 μM, as well as a TRPV1 agonist (EC50 value between 1.0 and 2.0 mM) [27]. The TRP channel agonists (TRPA1 and TRPV1) facilitate a transient increase in the intracellular calcium ion concentration, likely indirectly affecting SGLT2 transport kinetics and/or expression levels [58]. However, the extent to which TRP channel agonism influences renal glucose transporter activity (SGLT2/GLUT2) remains undetermined since there are overlapping expression levels of TRP channels, cannabinoid receptors, and renal glucose transporters. These overlapping expression levels increase the uncertainty about which molecular mechanisms are responsible for the purported changes in renal glucose uptake. The in vitro findings on CBG’s diverse receptor and ion channel modulation, while not providing direct SGLT2 inhibition data, are highly relevant. They suggest indirect pathways for influencing renal glucose handling and overall metabolic balance, distinguishing phytocannabinoids from the singular SGLT2 targeting of synthetic inhibitors.

Aside from the cannabinoid and TRP channels described above, it appears that CBG displays pharmacological activity for various TRP channel and cannabinoid receptor isoforms: CBG (IC_50_ = 0.16 μM) inhibits TRPM8 and may also activate the TRPA1 (EC50 = 3.4 μM) and TRPV1 channels (EC50 between 1.0 and 2.0 mM), indicating a diverse pharmacological profile for CBG [27]. It can be assumed that the impact of the CBG cannabinoid receptor agonist/antagonist activity in renal proximal tubule cells may modulate intracellular calcium stores due to downstream events initiated by CB1/2 receptor activation [50]. This shift in intracellular calcium stores may indirectly alter intracellular transport kinetics, thereby impacting SGLT2-mediated glucose reabsorption.

Regarding direct agonism/antagonism of TRP channels within the renal proximal tubule and its impact on SGLT2, it is possible that phytocannabinoids may alter the transporter activity and/or expression level. Interestingly, similar overlapping expression levels of TRP channel isoforms are also noted for cannabinoid receptors and glucose transporter isoforms (SGLT2, GLUT2) within both renal proximal tubule and islet β-cells [59]. Although no evidence is reported indicating that CBG directly agonizes or antagonizes TRP channels on the membrane of the renal proximal tubule, an increase in the intracellular calcium concentration via other TRP channels like TRPA1 may also have the indirect consequence of affecting renal glucose transporter activity or expression by interfering with intracellular calcium-dependent enzymes that regulate glucose transport [60].

In vitro evidence has also indicated that THCV has the ability to restore insulin signaling and enhance glucose uptake [31], in addition to its hypoglycemic effect in vivo. Therefore, given the previous evidence mentioned throughout this paper of insulin modulating SGLT2 activity, it is possible that THCV, similarly to the plant extract described above, may directly alter SGLT2 protein or transport activity while simultaneously enhancing glucose reuptake through insulin receptor-mediated signaling [61]. By restoring the downstream mediators of insulin (IRS-1, AKT, and p70 S6K), the renal proximal tubule may indirectly express and/or transport glucose through the receptor and the SGLT2 protein.

Overactivation of CB1 receptors stimulates GLUT2 gene expression, leading to increased glucose reabsorption in rat renal proximal tubule cells in vitro [62]. Therefore, CB1 receptors may indirectly impact SGLT2 activity. CB1 overactivation by Δ9-THC also causes kidney dysfunction, yet CB1 receptor inhibition can reverse this by normalizing GLUT2 activity [63]. CB1 antagonists can reduce the glucose uptake of renal proximal tubule cells by inhibiting GLUT2 expression through ERK1/2 phosphorylation inhibition [30]. However, no in vitro or animal studies have indicated that selective CB1 receptor agonists can directly or indirectly influence SGLT2 activity. Further experimentation is needed to elucidate the signaling pathways through which CB1 agonism may influence renal glucose transport.

CBG, through the binding of multiple TRP channels and the activation of calcium receptors, is believed to influence glucose transport mechanisms [64]. However, this is a pharmacological assumption given that there are various molecular receptors for calcium and calcium-dependent enzymes that play a role in cellular glucose management. Furthermore, in vitro studies have also indicated that high doses of CBG may influence insulin signaling in hepatoma and myotubes via insulin receptor kinase activity [31]. Nonetheless, more experimentation is necessary to validate whether phytocannabinoid modulation of glucose transport involves cellular mechanisms in addition to insulin receptor signaling or Ca2+ receptor signaling.

Although various publications have described binding interactions and subsequent pharmacological effects of plant-derived CB1/2 receptor agonists and antagonists, as well as the TRP channel agonists and antagonists mentioned above, no experiments have directly assessed and documented the extent to which the compounds influence in vitro glucose uptake in renal proximal tubule cells. Future in vitro studies should focus on performing assays to directly measure cellular SGLT2 inhibitory activity, ideally in various in vitro (e.g., SGLT2 protein expression levels on cell membranes, radioactive/fluorescent tracer-based cellular glucose transport, electrophysiological measurements) and in vivo models of type 2 diabetes. Ideally, the inhibition potency for SGLT2, for each active molecule, must be characterized quantitatively in appropriate animal cell models (e.g., IC_50_ values). Such studies would require the isolated phytocannabinoid, at varying concentrations, to be delivered to renal proximal tubule cell cultures. The in vitro and in vivo glucose transport effects of the active phytocannabinoid will likely vary depending on the in vitro cell type/organ. Nonetheless, the results will establish if there exists a pharmacological influence of phytocannabinoids on SGLT2 glucose uptake and inhibition.

### 5.3. In Vivo Evidence

**Figure 6:**
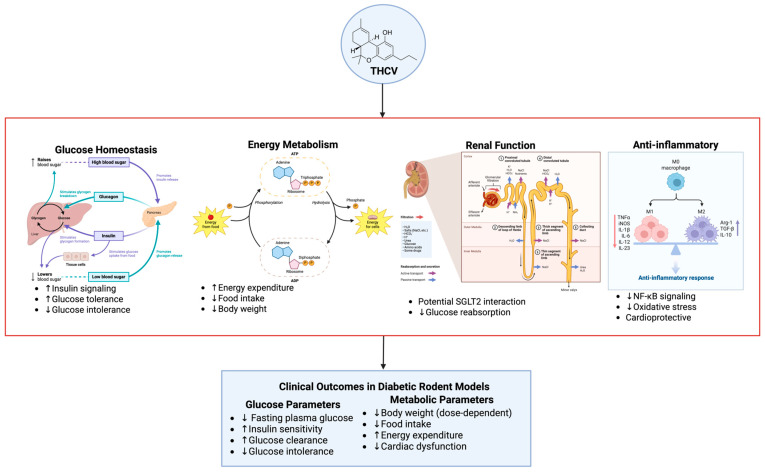
In vivo efficacy of THCV in diabetic rodent models. ↑: Increase/Rise; ↓: Decrease/Drop.

In vivo studies showed that phytocannabinoids could be helpful in ameliorating T2DM and diabetic complications [65,66,67]. The in vivo data are particularly valuable as they provide a more comprehensive physiological context for the potential of phytocannabinoids for managing T2DM, including aspects directly relevant to SGLT2 function. THCV showed glucose-lowering effects by stimulating energy expenditure and reducing glucose intolerance (Figure 6). These effects may contribute to reduced glucose reabsorption. Because sodium–glucose cotransporter 2 (SGLT2) inhibitors also diminish renal glucose reabsorption [31], the role of phytocannabinoids and, in particular, THCV must be explored for potential synergy with these drugs.

It has been shown that THCV restores insulin signaling in hepatocytes and myotubes of ob/ob mice [31], suggesting that THCV’s benefits extend to other targets associated with T2DM. Given that SGLT2 transporters are upregulated under systemic hyperglycemia and insulin resistance, and that THCV improves these, it may be useful to analyze its effect on renal glucose reabsorption in the setting of elevated serum glucose and insulin. A better comprehension of THCV’s mechanisms in regard to renal glucose homeostasis may bring insight into novel SGLT2 inhibitor escape mechanisms and potential future combinatorial strategies to bypass their development.

THCV was shown to elicit dose-dependent reductions in food intake and body weight in rodent models [31], whose metabolic profile resembles the moderate weight loss seen with SGLT2 inhibitor administration. Because obesity is a common comorbidity in patients with T2DM, the ability to achieve combined weight loss and glycemic control with phytocannabinoid use may be a significant benefit of this class of drugs. Further studies need to elucidate the central and peripheral mechanisms (s) mediating the food-intake- and weight-lowering properties of THCV and to verify if these effects are independent of glucose regulation. In the case where THCV effects are mediated through pathways distinct from those directly influencing glycemia, combinatorial benefit may still be attained if these effects synergize with the weight loss produced by SGLT2 inhibitors.

Improving glucose tolerance and insulin sensitivity with THCV, and the observation that increased blood glucose levels correlate with elevated glucose transport via SGLT2 [31], suggests the need to explore these improvements in the context of SGLT2 inhibition. Thus, THCV’s impact on systemic glucose clearance and how this influence can supplement that of SGLT2 inhibitors by relieving the burden of systemic hyperglycemia on SGLT2 activity remain important topics to explore. The possibility of providing dual mechanisms of reduced blood glucose via phytocannabinoid treatment, by both improved insulin sensitivity and glucose clearance as well as decreased SGLT2 transporter activity and renal glucose reabsorption, could serve as a comprehensive diabetes treatment. However, the ability of THCV to directly influence SGLT2 transporters to decrease glucose reabsorption remains underexplored.

The metabolic effects of THCV, including its effects on energy expenditure, weight control, and improved glucose and insulin regulation, may act in a synergistic fashion with the actions of SGLT2 inhibitors [31]. A clear rationale has been established for exploring these strategies in vivo, where experimental outcomes are more realistic than those achieved in vitro and in cell culture, and where experimental outcomes can be translated for future potential for clinical application. However, these in vivo studies are only helpful in validating novel concepts; there is no comparative data to support THCV as a superior option in the management of T2DM versus available SGLT2 inhibitors.

Studies in diabetic animal models have shown that treatment with CBD significantly attenuates cardiac dysfunction [68]. Cardiac output was improved by CBD treatment, and significant improvements were made in multiple markers of myocardial damage, suggesting a potential role for CBD in improving cardiac health under diabetic conditions, beyond solely addressing glucose levels [69,70,71]. The reduction in oxidative and nitrative stress in response to CBD treatment, as well as reduced myocardial fibrosis [68], shows the potential of CBD in treating specific pathologies associated with diabetic cardiomyopathy. While these CBD-mediated improvements of cardiovascular health appear complementary with the effects of SGLT2 inhibitors, the potential combined effects for improving systemic metabolic health have not been evaluated in vivo.

In addition to the reduction in stress within cardiac tissue, the decreased activity of the NF-κB signaling pathway in response to CBD treatment suggests a general anti-inflammatory response to CBD [68]. While SGLT2 inhibition has a more isolated mechanism involving renal glucose control, the broad anti-inflammatory properties of CBD could offer an improved overall therapeutic effect when used as a combination therapy for T2DM. However, the effect of long-term administration of CBD in populations susceptible to inflammation-induced diseases, such as those with existing cardiovascular or kidney impairment, needs to be examined, as these groups may have a reduced threshold for drug-induced inflammation.

Apoptosis of cells within cardiac tissue was reduced in response to CBD treatment [68]. Unlike the effects of SGLT2 inhibitors, which mediate primarily urinary glucose loss, the reduction in organ damage on a cellular level indicates that CBD can promote improvement of systemic diabetic symptoms [72]. As a result, an in vivo investigation is needed to fully validate CBD as a combinational strategy for SGLT2 inhibitors in order to obtain synergistic reduction of overall disease severity.

With the dose-dependent reductions in food intake and body weight induced by THCV [31], in vivo testing may confirm the possibility of improving T2DM symptoms, as well as controlling associated conditions like obesity and cardiovascular damage, with a single phytocannabinoid-based therapy. The exploration of these multi-targeted drugs versus the use of the more isolated mechanisms of conventional anti-diabetic strategies can be an effective method for improving current therapies for T2DM and for developing novel future drugs with synergistic multi-target benefits. Pharmacodynamic evaluations are needed to more fully elucidate the interplay of the metabolic effects of THCV with the ability to impact renal glucose transport in the context of systemic hyperglycemia and/or hypoinsulinemia.

THCV, as well as CBD, may elicit beneficial changes in glucose regulation and control of associated metabolic dysfunction without inducing many psychoactive side effects, as was the case for the earlier synthetic CB1 antagonists that were withdrawn from clinical trials for this reason [30]. As a result, the possibility to use phytocannabinoids as a safer alternative to target components of the peripheral endocannabinoid system as a novel therapeutic for metabolic disorders has gained increased support in recent clinical and preclinical studies. In this context, these studies must determine whether the proposed benefits of phytocannabinoid administration can achieve the desired clinical goals for these patient groups, with equivalent safety in comparison to other currently available treatments.

There is growing evidence in the literature that supports a potential for regulating glucose homeostasis by modulating the activity of the peripheral endocannabinoid system [30]. The precise crosstalk mechanisms between these CB receptors and other membrane receptors or transporters, such as SGLT2, need more elucidation and remain an important question to address for future directions. These findings can reveal how these mechanisms of action could potentially be co-exploited for improved treatment of metabolic disorders like T2DM.

Additionally, indirect beneficial actions of phytocannabinoids in vivo, such as their anti-inflammatory, cardioprotective, and antioxidant functions [30,68], may serve to combat various diabetic complications such as cardiovascular disease and chronic kidney injury, resulting in a lower incidence of comorbidities to improve the overall treatment strategies for patients with T2DM.

In vivo studies show that phytocannabinoids possess a distinct ability to act in concert with synthetic SGLT2 inhibitors to promote a synergistic outcome in the combined effect [30]. Further experiments and models that expand on this hypothesis can provide meaningful insight into these interactions that will be important in the development and refinement of future diabetes therapy options.

Although substantial information regarding the beneficial effects of phytocannabinoids for treating diabetes and its comorbidities has been gathered in vivo, little remains known of the detailed mechanisms by which they can alter the cellular components that are thought to be responsible for those beneficial effects, and no clinical data is yet available. Further work is needed to evaluate their potential for effective SGLT2 regulation and combination with current anti-diabetic strategies.

The in vivo data on THCV and CBD thus represent highly relevant and original contributions, showcasing their potential to offer a more holistic approach to T2DM management compared to the more singular action of synthetic SGLT2 inhibitors. These findings are crucial in building the rationale for combining phytocannabinoids with or developing them as alternatives to current SGLT2-based therapies.

## 6. Clinical Efficacy and Therapeutic Potential

From a real-world perspective, the potential of phytocannabinoids to manage type 2 diabetes will be looked at in relation to clinical evidence, therapeutic benefits, and safety considerations. Their ability to provide relief and improvement in the management of this condition will also be compared to already established synthetic SGLT2 inhibitors.

### 6.1. Clinical Studies and Case Reports

Clinical studies for THCV in the context of type 2 diabetes mellitus (T2DM) show potential benefits in glycemic control. It has been discovered that administering THCV to 62 diabetic patients causes a decrease in fasting plasma glucose levels and improved pancreatic β-cell function [31]. These clinical observations directly supported THCV’s efficacy in restoring insulin signaling and enhancing glucose uptake in insulin-resistant animal models, highlighting a clear translational relevance from preclinical data to clinical potential. The reduction in fasting plasma glucose levels is clinically helpful as it is an important element in diabetes management. As such, THCV affects endogenous insulin function, as opposed to SGLT2 inhibitors, which can only be used at the periphery of pancreatic islet systems [73]. Thus, phytocannabinoid treatment in T2DM can become superior compared to synthetic inhibitor drugs. Larger sample studies still have to be completed to solidify these claims, since the current sample study consisted of only 62 individuals.

THCV displays dose-dependent improvement in glucose tolerance and insulin sensitivity in vivo [31], similar to in vitro studies. This is helpful because it portrays the reliability of THCV’s metabolic function. This reliability means that translational work into clinical cases would be useful; however, as mentioned, larger sample sizes for these clinical studies must be completed in order to ensure safety.

The increased functioning of pancreatic β-cells implies that THCV not only works as a renal glucose reabsorption agent. Improved functionality of β-cells means that THCV aids in insulin secretion and supports pancreatic health in order to control the overall blood glucose levels of T2DM patients [31], whereas synthetic SGLT2 inhibitors induce glucose excretion. Thus, THCV displays a multifaceted mechanism to alleviate T2DM that allows for greater potential in T2DM therapeutics. Still, these findings must be expanded upon to show long-term benefits to β-cell function, and systemic metabolic pathways should also be accounted for.

Reduced levels of fasting plasma glucose are another finding that indicates a positive association between THCV and T2DM. This outcome alone shows potential for the compound to be a useful pharmaceutical intervention to help combat T2DM; however, because data are mostly from pilot studies [30,31], it is important that larger studies occur to validate these claims. Moreover, a detailed description of dosing studies and subgroup analysis should be completed in order to optimize the benefits of combating T2DM.

Because psychoactive and psychiatric side effects have not yet been documented in current data, in contrast to what was found in synthesized cannabinoid antagonists, THCV demonstrates great therapeutic potential against T2DM [30]. This favorable clinical safety profile for THCV and other phytocannabinoids, such as CBD, is a key distinguishing feature, directly correlating with relevant in vitro and in vivo mechanistic studies showing their selective action and lack of significant central psychoactive effects, unlike problematic synthetic CB1 antagonists. It is important to remember that the synthetic CB1 antagonist rimonabant was removed from therapeutics because of its strong association with psychiatric disorders such as depression. In addition, because many living with T2DM suffer from depression [30], it is highly important to avoid inducing these side effects during pharmaceutical intervention for the disease. As such, more data needs to be acquired, especially data showing chronic tolerability of THCV and adverse effects.

Participants receiving THCV exhibited a reduction in subjective hunger. These initial observations are helpful to explore the use of THCV as an intervention to not only regulate blood glucose levels, but also regulate body weight [31], seeing as patients suffering from T2DM show a high risk of being overweight or obese [30]. The cause of decreased hunger can likely be attributed to THCV’s CB1 receptor antagonistic activity. Because THCV shows CB1 antagonism, it is considered an anorexigenic phytocannabinoid, as opposed to tetrahydrocannabinol (THC), which stimulates hunger and can therefore be considered an orexigenic cannabinoid [30]. Thus, further research must be completed to show that THCV reduces hunger and to evaluate what cellular pathway mechanisms explain this phenomenon.

Furthermore, one of the greatest issues in the therapeutics of synthetic cannabinoids was the neuropsychiatric side effects. For example, several CB1 antagonists were pulled from shelves because of adverse psychotic behaviors [30]. As such, it must be indicated that psychoactive or psychiatric side effects were not observed throughout the THCV study. THCV and other phytocannabinoid investigations are a far leap in this field in comparison to previous synthetic cannabinoid studies for metabolic therapeutics because they demonstrate a relatively safe impact [31]. Although positive outcomes were portrayed in these studies for the effectiveness of treating metabolic diseases using cannabinoid interventions, chronic treatments and their impact on mental health were not thoroughly assessed, and thus it is imperative to further evaluate the neurological consequences of cannabinoid usage, such as THCV and CBD, for T2DM therapies in clinical trials.

The fact that there were no changes to anxiety scores on scales between participants taking THCV and placebo may indicate the potential use of THCV as a pharmaceutical option to increase insulin sensitivity in patients [74]. However, there are still limitations in this area that need to be addressed in future studies and designs for clinical trials, since it has to be clarified whether these effects in blood sugar are more closely aligned in their nature with animal studies that used high dosages of THCV [31] for extended periods in order to reduce hyperglycemia and improve glucose resistance and responsiveness. More data would be needed to confirm if it works similarly in humans; thus further research would need to be developed to include animal and human trials to compare and see the dose-dependent response as well as the length of its effect.

Comparing phytocannabinoids and synthetic CB1 receptor antagonists would be useful to describe any differences in patient tolerability. Rimonabant, a synthetic CB1 antagonist, had negative psychiatric side effects that resulted in the withdrawal of the therapeutic drug. Since then, phytocannabinoids such as CBD and THCV have been able to bypass these neuropsychiatric side effects in early studies [30]. In addition to the lack of psychiatric events, cannabinoids have been well tolerated, without elevated levels of anxiety, depression, or psychosis. The reason why synthetic forms induced such effects in patients is that they were agonists of the CB1 receptors that were located in the CNS [30], while THCV did not show such activity because it showed its antagonistic influence at the CB1 receptors and had a dose-dependent response in decreasing fasting plasma glucose, which is clinically applicable. This shows that the benefits can still be gained from CB1 activation while there are no neuropsychiatric side effects, resulting in no difference compared with the placebo treatment. But there are also effects on improving insulin sensitivity in humans [31] and on improving glucose responsiveness and resistance to insulin in animal models. Since this is not well understood in its mechanism, it would need to be addressed and examined further with the dose responsiveness.

The notion that cannabinoid receptors, if activated, are guaranteed to display psychoactive events can be dispelled through examining CBD and THCV. These compounds demonstrate potential candidates for chronic metabolic therapeutics due to a reduced association with psychiatric side effects and elevated benefits in T2DM treatment [30]. However, clinical trials are still required to confirm these statements for use in practical applications.

It should be noted that in recent case reports, β-caryophyllene, which has shown CB2 activity and is a cannabinoid often found in foods and herbs [52], also displays antihyperglycemic activity that is accompanied by organ-protective activities and anti-inflammatory activity. These real-world observations on BCP’s observed benefits in hyperglycemia, inflammation, and organ protection underscore the clinical relevance of original in vitro studies that identified its CB2 agonist and PPAR activator properties, suggesting a broader, multi-targeted therapeutic action beyond direct SGLT2 inhibition that complements traditional diabetes management strategies. Interestingly, it may also exert its effects by regulating the expression of PPARs [52]. These properties can allow β-caryophyllene to combat many complications of T2DM in an effective manner, such as inflammation and renal damage, as well as hyperglycemia. These properties can be effective to study in order to test novel therapeutics for T2DM patients. Therefore, because of the organ-protective properties, antioxidant and anti-inflammatory activities, along with the β-caryophyllene showing to regulate the expression of PPARs [52], it shows to be a novel anti-diabetic therapeutic with a protective effect.

The shift in perspective towards phytomedicinal therapies that mimic SGLT2 inhibitors, while also improving complications like diabetic nephropathy [55], can be explained by positive translational and clinical studies thus far that present effective and relatively safe treatment in comparison to synthetic cannabinoid therapeutics. Because of their tolerability and low incidence of psychoactive side effects, this further emphasizes the positive and emerging evidence. Although the preliminary studies support the use of phytomedicine for T2DM, the most difficult issue facing the approval and acceptance of plant-based interventions is quality and standardization. Thus, more evidence should be supplied with quality trials in order to address these concerns, especially for phytocannabinoids, because they can be complex and not widely understood in terms of what they may accomplish and their efficacy in treatment, although studies are increasingly available for investigation, and the need for them is important in order to understand this.

Moreover, these results can encourage cannabinoid therapies for T2DM. Although there may be varying degrees of effectiveness and tolerability amongst phytocannabinoids, they show some potential as monotherapy options or adjunct interventions. Thus, larger studies need to be conducted to evaluate their comparative value.

In any case, these challenges in phytocannabinoid therapies are not necessarily discouraging factors that cause them to fail. Still, it shows to be a novel approach as evidence continues to grow to address these major research and regulation gaps. In conclusion, these studies display a growing emphasis that THCV, along with many other plant-based cannabinoids such as CBD, may serve as useful interventions for T2DM. By decreasing fasting plasma glucose levels and stimulating insulin sensitivity in diabetic patients, thus having the potential to combat T2DM in ways similar to the pharmaceutical intervention of SGLT2 inhibitors in helping to reabsorption glucose from the kidneys, these agents offer great potential in this field.

### 6.2. Comparison with Synthetic SGLT2 Inhibitors

The development of synthetic SGLT2 inhibitors has offered highly potent and selective inhibition of the sodium–glucose cotransporter 2 (SGLT2) through agents such as dapagliflozin and canagliflozin. These molecules boast IC_50_ values in the nanomolar range and therefore effectively limit renal glucose reabsorption in the kidneys of T2DM patients [23]. Novel analogs compound 2 and compound 6 have achieved potent SGLT2 inhibition through structural optimization, resulting in 0.64 and 4.5 nM, respectively, and enhanced SGLT2 selectivity [23]. In contrast to these established synthetic compounds with precise, quantifiable SGLT2 inhibition, original in silico docking studies have provided crucial and relevant initial insights, revealing that several phytocannabinoids, including THCV and CBG, exhibit high binding affinities for the SGLT2 substrate pocket, suggesting a novel direct modulatory potential that warrants further quantitative in vitro and in vivo validation. These advances in SGLT2 inhibitor potency and selectivity for synthetic molecules have improved clinical outcomes. However, a downside to targeting a single pathway is that these drugs have little to no impact on many other factors that contribute to the development of the disease (SGLT2 alone).

Structural optimization has led to long-acting and intermediate-acting SGLT2 inhibitors, ensuring that drugs are able to offer consistent therapeutic effects for predictable durations [75]. This has drastically improved drug tolerability through ensuring drug stability and controlled drug release in a consistent and reproducible manner. This highlights the technological sophistication in synthetic molecules, while, for example, some phytocannabinoids offer only long-acting inhibition, with their effect being restricted only to glucosuria or even only to one type of glucosuria.

Select phytocannabinoids tetrahydrocannabivarin (THCV), cannabidiol (CBD), and cannabigerol (CBG) exhibit similar, but not measured, in silico and in vitro SGLT2 inhibition efficacy in renal glucose handling as synthetic molecules [19,27]. However, these molecules have yet to be confirmed as SGLT2 inhibitors via direct measurements such as IC_50_ values. This makes it difficult to assess SGLT2 binding and, thus, difficult to gauge their relative strength as SGLT2 inhibitors when comparing them with synthetic drugs that can provide these values. Unlike synthetic compounds that have SGLT2 as their sole target, several phytocannabinoids have a much broader polypharmacological effect, impacting a multitude of TRP channels and PPARs [27]. Therefore, these molecules are able to interact with a wide variety of therapeutic targets in T2DM. While this may offer an advantage in tackling this disease with a multibranched etiology, such as T2DM, which is a multi-target disease, this could make predicting overall effects extremely challenging.

Synthetic SGLT2 inhibitors lower glucose primarily through promoting glucosuria, and in this way, these molecules are effective at lowering plasma glucose concentrations without inducing hypoglycemia because they act independently of the need for the pancreas to secrete or respond to insulin [75]. However, most phytocannabinoids appear to have the capacity to not only impact the kidneys through the SGLT2 receptor by inhibiting glucosuria but also have the potential to impact insulin secretion and insulin sensitivity, lower glucose intolerance, increase energy expenditure, and more [30,31]. Therefore, these compounds may have the opportunity to impact T2DM by acting through multiple mechanisms, both systemically and at the kidney SGLT2 receptor level.

Preclinical and clinical studies have shown that the pleiotropic profile of some phytocannabinoids demonstrates anti-inflammatory and antioxidant activity, which in turn can protect organs against the long-term damage caused by T2DM [30,68]. The beneficial effect on organ protection observed in these studies demonstrates a notable advantage for phytocannabinoids over conventional synthetic SGLT2 inhibitors in treating diabetes-related complications, such as chronic inflammation, chronic oxidative stress, and CVD. While these studies demonstrate promising therapeutic benefits for phytocannabinoids, they are preclinical, and the therapeutic benefits must be validated by conducting systematic human trials to have an impact on the outcomes for people living with T2DM.

Dapagliflozin and ipragliflozin are long-acting SGLT2 inhibitors and are taken by patients on a daily basis [75]. These molecules are stable and reliable at improving pharmacokinetic parameters. This ensures a predictable pharmacokinetic profile and, in turn, provides patients with a consistent effect that can be reliable, regardless of background genetics. In contrast, most phytocannabinoids are poorly water-soluble, undergo extensive first-pass metabolism, and exhibit inter-individual variability in systemic absorption and exposure, and therefore may not be the most robust, reliable option when compared to synthetics in achieving effective SGLT2 inhibition [33]. Although these challenges hinder the effectiveness of cannabinoids in reaching the clinic, several methods, such as self-emulsifying drug delivery systems, have been implemented for the efficient oral delivery of cannabinoids and enhancing their bioavailability to improve their pharmacokinetic profiles and clinical effectiveness [33].

Synthetic SGLT2 inhibitors have shown effective reductions in HbA1c levels, along with a reduction in diabetic nephropathy and cardiovascular events in both rodent and human studies, as well as providing other clinical benefits. However, synthetic SGLT2 inhibitor treatments come with side effects, the most prominent being genitourinary infections, as well as an elevated risk of developing euglycemic ketoacidosis, a particularly dangerous complication [75,76]. Some studies have reported that THC and other cannabinoids can have side effects that include psychoactive/psychiatric abnormalities [77,78]; however, some of these results can be attributed to differences between cannabinoid types used in the studies. In contrast, the preclinical and early clinical data for a variety of cannabinoids, such as THCV, CBD, and CBG, do not result in psychoactive or psychiatric effects, even in long-term exposure studies, and it is also documented that these compounds counteract some side effects of THC [30,31]. A major component in designing new therapeutic strategies in phytocannabinoids is to avoid the side effects observed with THC and maximize the beneficial effects of other phytocannabinoids. Thus, phytocannabinoid-based therapeutic applications appear to have an extremely favorable safety profile compared to synthetics and potentially offer the opportunity to serve as a safer therapy for people with diabetes.

For future applications, the rational development of phytocannabinoid therapeutics may have the ability to outperform the potency of synthetic drugs at the SGLT2, as well as avoid some of the side effects and provide multiple benefits that were discussed previously [31,55]. This can be accomplished through structure–activity relationship studies (SAR) to maximize binding affinity of specific molecules and/or molecular targets, as well as to limit negative side effects through molecular design. In addition, the performance of phytocannabinoid-based therapeutics can be compared directly to existing synthetic SGLT2 inhibitor medications, and the comparison may suggest additional targets to explore to alleviate T2DM that synthetic SGLT2 inhibitors are not capable of targeting [79].

Combination therapies may be designed with the objective of supplementing the effects of each individual molecule, with the aim of improving the therapeutic value by treating a greater number of conditions or treating a single target with greater efficacy when single therapeutics fall short [55]. For instance, phytocannabinoids can be combined with other glucose-lowering agents such as synthetic SGLT2 inhibitors to improve patient outcomes, such as using THCV with synthetic SGLT2 inhibitors to increase insulin sensitivity and glucose reabsorption.

To conclude, synthetic SGLT2 inhibitors have strong and potent affinity for SGLT2, as well as efficacy for glucosuria, which can result in effective control over blood glucose levels. However, as effective a treatment as this is, it comes with potentially concerning side effects, which the phytocannabinoids do not possess. These phytocannabinoids, while not as efficacious in humans at this time, still have the potential to impact glucose levels in the human kidney, in addition to having the potential to improve or treat a variety of health problems through their multiple-target ability.

### 6.3. Safety Profile and Adverse Effects

Phytocannabinoids such as CBD and THCV appear to possess an improved safety profile regarding psychoactive or psychiatric side effects in comparison to the synthetic cannabinoid receptor antagonist rimonabant [30]. CBD and THCV have been evaluated in both preclinical and clinical settings without inducing depression or anxiety, which can arise as a result of the CB1 receptor blockade by rimonabant [30]. The relative neuropsychiatric safety of these phytocannabinoids may be attributed to their receptor selectivity, as CBD acts as an antagonist or negative allosteric modulator on CB1 receptors. Another factor supporting the neuropsychiatric safety of phytocannabinoid therapies is that CBD does not cross the blood–brain barrier (BBB) as readily as Δ9-THC [80]. By acting on CB1 receptors through different mechanisms, phytocannabinoids bypass some of the adverse effects associated with psychoactive cannabinoids that broadly activate cannabinoid receptors [30]. Still, large-scale trials evaluating long-term neuropsychiatric safety need to be conducted on phytocannabinoids.

Preclinical data indicate that CBD and THCV improve metabolic syndrome and reduce insulin resistance in mice when applied through peripheral endocannabinoid receptor stimulation [81,82]. Specifically, THCV reduced adiposity, improved hepatic fat metabolism, and enhanced insulin signaling in both adipocytes and hepatocytes in diet-induced obesity models, while CBD reduced adipogenesis and improved insulin signaling in cultured adipocytes [30]. These phytocannabinoids may improve energy homeostasis through peripheral pathways by minimizing central neurobehavioral effects [83]. Limited human studies provide evidence that several plant-derived cannabinoids exert a high tolerability and negligible psychiatric side effects up to doses 10 times above what is considered clinically relevant [30]. Together, preclinical and early human trial data support the use of phytocannabinoids as promising therapies that improve diabetes-relevant metabolic abnormalities. Further clinical evaluations are needed to determine if the relative neuropsychiatric safety seen with phytocannabinoids can translate into clinical benefit.

Renal safety of phytocannabinoids appears to be somewhat complex. Evidence suggests that CB1 blockade improves diabetic nephropathy and reduces oxidative stress and inflammation in the kidney in the presence of diabetes [84]. CB1 stimulation can exacerbate nephropathy as a function of the disease context and dosages [30]. While β-caryophyllene shows beneficial effects in rodent kidneys upon CB2 stimulation, it is not clear how these findings can translate to humans, suggesting that careful assessment is necessary [30]. There needs to be a mechanistic assessment of the impact that phytocannabinoid administration has on the kidneys in the context of diabetes. Long-term safety data are necessary in order to assess the viability of this type of treatment.

The major weakness in phytocannabinoid therapies is the lack of well-controlled pharmacokinetic behavior. In contrast to synthetic drugs like SGLT2 inhibitors, the oral bioavailability and pharmacokinetics are not always highly reproducible. Pharmacokinetics for phytocannabinoids like CBD are relatively unknown and variable because of CBD’s poor solubility and high pre-systemic metabolism in the intestine and the liver. This results in oral bioavailability between 6 and 20 percent [33]. It can also be difficult to estimate suitable doses of CBD because they are so greatly impacted by inter-individual differences in absorption. Furthermore, it is unclear how CBD serum levels can influence bioavailability, leading to erratic exposure levels. CBD therefore requires extremely high doses, between 20 and 30 mg per kilogram, which can lead to adverse effects. The highly variable oral bioavailability of phytocannabinoids leads to an increased risk of suboptimal drug exposures that fail to provide clinical benefit as well as the potential for supra-therapeutic or toxic effects [85,86]. Given the low therapeutic window for these plant-derived medicines, these pharmacokinetic limitations can preclude their usability for glycemic control to the extent that oral doses must be frequently adjusted to minimize the probability of dangerous and erratic alterations of glucose reabsorption [33]. Newer solid-state CBD formulations, in combination with self-emulsifying drug delivery systems, are currently undergoing clinical validation to improve oral absorption and bioavailability, but they have not reached their final application for this therapy for T2DM [33].

Phytocannabinoid polypharmacology suggests there are potential health benefits in addition to targeting diabetes-related pathology, but there are also safety issues to consider. In addition to CB1 and CB2 cannabinoid receptors, CBD is also known to have diverse effects at over 60 distinct targets within the human genome [30]. Many of these targets relate to metabolic activity, and this would allow the benefits of phytocannabinoid therapeutics to extend beyond their main indication for T2DM. These include anti-inflammatory, antioxidant, cardioprotective, and antineoplastic effects [87,88]. Despite the potential to impact various physiological processes, there are off-target effects that may arise from polypharmacology. The stimulation or inhibition of other targets of phytocannabinoids may elicit unexpected toxicities or adverse health outcomes. CBD interactions with TRP channels and PPARs have not been fully explored for their impact on the renal glucose reabsorption mechanism and whether that impacts kidney structure, physiology, or pathology in the context of diabetes. In particular, phytocannabinoid effects on renal hemodynamics, glomerular filtration rate, and tubular reabsorption rates should be closely monitored, especially considering that both CB1 and CB2 receptors play a role in these processes [84]. Also of particular importance is the possibility for interactions between phytocannabinoids and other SGLT2 inhibitors used in treatment for T2DM. All of these factors point to a significant necessity for rigorous safety monitoring when using this particular therapeutic. Some of the key markers that should be carefully examined include, but are not limited to, the impact on other bodily organ systems. These include hepatic, cardiac, and immune system markers, just to name a few. If CBD therapies have a practical place in the treatment for T2DM, comprehensive safety monitoring will need to be employed.

The wide variance in cannabis extracts as well as delivery systems greatly impacts the effectiveness of CBD therapies. *C. sativa* has great strain-level differences with variable ratios of cannabinoids that can significantly impact the therapeutic and/or adverse effects that patients experience [33]. The strain-level variation in chemical profiles in cannabis raises concern that chemovars (chemical varieties) of *C. sativa* that show positive therapeutic results will not translate to other chemovars, even when cultivated with the same protocols or when used on patients for the same indications. Some concerns about this have been allayed as there have been reports on chemovars that are comparable regarding phytochemical levels; however, concerns about chemovar-level variation remain an important area to be addressed and regulated. Additionally, differences among cannabis plant preparations or extractions can also introduce significant variances. Oral bioavailability varies depending on dose strength, the use of single cannabinoid molecules vs. chemovar extracts, and chemovar phytochemical ratios, as well as formulation. Another concerning factor is that current cannabis extractions do not have a high standardization across manufacturers for purity or concentrations of different molecules of interest, such as CBD and THC [33]. Even the variability of these medicines when obtained within the same batch of products from the same producer presents risks of suboptimal exposures. These variations are further exacerbated by the fact that different patients have highly variable pharmacokinetic behavior regarding all cannabinoids. Several efforts are undergoing to standardize cannabinoid extractions to improve the reliability of therapeutic effectiveness. Pharmaceutical manufacturers are in the early stages of development of formulations that combine CBD or combinations of cannabinoids with well-validated methods of delivering pharmaceuticals. Some of the ways being evaluated include delivery via oral ingestion of solid-state pills or capsule formulations, as well as use of self-emulsifying drug delivery systems (SEDDS). These solid dosage forms combine the ease of bioavailability of CBD while also maximizing exposure within the patient and reducing dose variability [33].

There has also been a move towards greater standardization of production to allow the reproducible assessment of benefits across manufacturers. Regulatory standardization efforts need to be in alignment in all areas, from the initial breeding of the *C. sativa* chemovars, the extractions of target compounds, all the way down to the final pharmaceutical formulation in solid or emulsion states. The standardization efforts must begin with improved identification and isolation of targeted chemovar variants for *C. sativa* that can have reproducible therapeutic effectiveness regarding indications for the desired disease state. This has begun by focusing on genomic-based methods of identifying different cannabis strains with a high level of reliability. Once these efforts succeed at allowing reliable detection of strains with well-defined cannabinoid contents, there will have to be effective development of growth and harvest protocols that result in reproducible concentrations of each desired cannabinoid in cannabis biomass. This will then enable the development of pharmaceutical preparation protocols, including extraction or total cannabinoid/phytochemical content per unit mass, as well as specific extraction processes. This is of utmost importance given the high variation in formulations across and even within cannabis producers. By achieving reproducibility of cannabinoid extraction in addition to improving extraction techniques, pharmaceutical companies can then start to focus on the final stages of pharmaceutical synthesis: the manufacturing of pills, capsules, emulsions, etc. These techniques will maximize cannabinoid exposure by the patient without inducing high variance in the absorbed dose [33]. However, without standardization efforts prior to this last stage, all cannabinoid pharmaceuticals will be significantly and negatively affected.

The benefits of including phytocannabinoids in the management of diabetes would be in counteracting several of the detrimental effects of T2DM, such as renal glucose over-reabsorption. In order for this treatment to be a valid alternative to synthetic pharmaceuticals used today, several of the pharmacokinetic and delivery-related challenges must be overcome. By improving this particular area of study of these plant-derived medicines, the full potential of them can be utilized to treat chronic diseases like T2DM.

## 7. Challenges and Development Considerations

Phytocannabinoids face significant bioavailability and formulation challenges, which inhibit them from becoming potential therapeutic agents for SGLT2 modulation in diabetes. Their oral bioavailability is poor, attributable to the hydrophobicity and limited solubility of compounds like CBD [89]. A large part of CBD, upon oral administration, undergoes extensive first-pass hepatic metabolism. Therefore, this leads to inconsistent systemic exposure and a lack of therapeutic predictability [33]. Inter-individual variability in absorption and metabolism also contributes to varying systemic exposures, which leads to variable clinical outcomes and necessitates individual dose optimization. In order to overcome such shortcomings in bioavailability, strategies like self-emulsifying drug delivery systems and solid-state formulations, etc., are employed to enhance solubility and bioavailability [33]. The aim of these delivery systems is to produce therapeutic levels, reduce the inconsistency in the patient-to-patient and patient-to-patient variability in the PK parameters, and ensure a consistent therapeutic response [90]. Nevertheless, most of these delivery strategies are still in the preclinical and clinical phases, and they still have various challenges in the large-scale manufacturing of pharmaceutical-grade formulations. Moreover, multi-component, phytocannabinoid-containing extracts come with challenges different from single-molecule, synthetic drugs, as the interaction between different phytocannabinoids and other natural compounds can result in further complexity regarding the pharmacokinetic parameters [91]. Thus, sophisticated pharmaceutical formulation and formulation science must be employed in order to optimize these delivery systems to ensure the activity and stability of phytocannabinoid mixtures.

Furthermore, a broader perspective on the existing limitations and criticalities of the current evidence underscores the substantial hurdles in the clinical translation of phytocannabinoid-based therapies. Despite promising preliminary findings, the current evidence base has several notable limitations that warrant critical discussion. Firstly, a significant portion of the data is derived from in silico and preclinical (in vitro and in vivo animal) studies, which, while valuable for mechanistic insights and lead compound identification, often face challenges in direct translational applicability to human physiology. Direct, quantitative measurements of SGLT2 inhibition by specific phytocannabinoids, such as IC_50_ values, are largely absent from in vitro studies, making direct comparisons with potent synthetic inhibitors difficult. Secondly, the inherent polypharmacology of phytocannabinoids, while offering pleiotropic benefits, also introduces complexity in elucidating precise mechanisms of action and increases the potential for off-target effects and drug interactions, which are not yet fully characterized. The interplay between SGLT2 modulation and the effects on other targets like cannabinoid receptors, PPARs, and TRP channels needs more thorough in vivo investigation to understand their integrated physiological impact. Thirdly, significant pharmacokinetic challenges, particularly poor oral bioavailability, extensive first-pass metabolism, and high inter-individual variability in absorption and exposure, severely limit the clinical utility and standardization of phytocannabinoid-based therapies. While novel delivery systems are being developed, their large-scale implementation and validated efficacy in human subjects are still in their early stages. Fourthly, the lack of standardized cultivation, extraction, and formulation protocols for *C. sativa* leads to considerable batch-to-batch variability in cannabinoid composition and concentration, compromising the reproducibility of research findings and the consistency of therapeutic outcomes. This necessitates rigorous quality control and clear regulatory frameworks, which are currently lacking. Finally, large-scale, well-controlled human clinical trials are notably scarce, with most clinical data stemming from pilot studies or case reports with small sample sizes. This limits the generalizability of findings and the ability to confidently assess long-term efficacy, safety, and comparative benefits against established synthetic SGLT2 inhibitors in diverse patient populations. The long-term safety profile, especially regarding potential neurological consequences and interactions with other medications, requires extensive investigation. Addressing these criticalities is paramount for the successful translation of phytocannabinoids into viable diabetes treatments.

The phytocannabinoid extract batch composition and concentration are responsible for influencing the therapeutic efficacy and safety of phytocannabinoid-containing treatments. The variation in cannabinoid composition and concentration is due to various factors like the genetic difference among various strains of *C. sativa*, environmental factors during the plant growth and harvesting, and inconsistent post-harvest processes and extraction techniques [33]. These variations among the batches are responsible for the inconsistencies in the preclinical and clinical studies and complicate the analysis of the pharmacodynamic properties. Variation in the cannabinoid composition may lead to difficulties in dose titration and may not yield the expected efficacy. The impact of the existence of uncharacterized minor cannabinoids and other co-extracted natural products on safety and efficacy is poorly understood; in certain cases, it can result in unpredictable adverse effects. There are also no standardized procedures for quality control in terms of purity, cannabinoid concentration, impurities, and presence of pesticides and other heavy metals. Thus, for the usage of plant-based medicines, these should be quantified with analytical techniques like HPLC and in accordance with GMP [33].

The lack of regulatory clarity stands out as one of the most critical challenges to the progress of phytocannabinoid therapies for the control of SGLT2 in diabetes treatment. Differences in laws across different regions regarding the cultivation, extraction, and medicinal use of cannabis impede the progress of phytocannabinoid therapy by posing several hurdles to its research. Only very few cannabis-derived products (e.g., Epidyolex™) have been approved for use by regulatory agencies, and in most of these cases, the use is limited to certain rare disease conditions rather than the treatment of more widespread chronic diseases like T2DM [92]. Thus, the restriction on the use of cannabis-derived pharmaceuticals has discouraged researchers from investing in this area. Furthermore, due to the unavailability of a clear approval path in different states and countries, there have not been many multicenter clinical studies on the therapeutic potential of phytocannabinoids. As a result, there is a shortage of large-scale, multi-ethnic human clinical research to support the use of phytocannabinoids in the treatment and management of various diseases. Due to the variation in regulatory structures, most phytocannabinoid products are not well regulated, and thus most of these phytocannabinoid-based supplements cannot be assumed to be of good quality, composition, or labeled with the correct dosage, therefore giving consumers the incorrect assumption.

The legal ambiguities and challenges surrounding cannabis for medicinal use vary from the state and country to country. To date, there are legal complications associated with cultivation, extraction, and use that contribute to delays in gaining ethical approvals and institutional review board clearances, etc., for the initiation of clinical trials with phytocannabinoid extracts [93,94]. As a result, it discourages pharmaceutical companies from undertaking and translating initial positive preclinical data into human studies [92]. In addition, there are also ethical issues related to the use of cannabis in certain vulnerable populations, such as children and those with mental health-related issues. Moreover, it has been recommended that a proper risk-to-benefit ratio should be studied before conducting studies on humans with cannabis extracts [92]. There are many unregulated phytocannabinoid-containing products available in the market with incorrect therapeutic claims, but these products lack adequate regulations of quality control, composition, etc., which poses risks to public safety.

The phytomedicine approach as a potential SGLT2 modulator brings certain practical challenges for the effective implementation of this strategy. Many in silico and preclinical studies suggest that phytocannabinoids like tetrahydrocannabivarin (THCV) exhibit moderate binding to SGLT2 and reduce renal glucose reabsorption [55]. However, the direct clinical confirmation of potent SGLT2 inhibition in the human body by the phytocannabinoid is minimal. The significant limitation of their bioavailability and variable pharmacodynamics further complicate the dose optimization. Furthermore, the quality control of plant extracts is always challenging because, from batch to batch, these cannot be standardized for their active contents. This poses a major barrier to achieving consistent formulation effects. The regulatory approval path for phytomedicines is not very well understood as compared to other therapeutic approaches. Thus, large-scale clinical evidence with robust evidence on pharmacokinetics, pharmacodynamics, efficacy, safety, and cost-effectiveness is required. Further clinical evidence needs to be generated, as most studies on the impact of *C. sativa* are characterized by varying degrees of heterogeneity, small sample sizes, and variable designs, and have not led to definitive results [55].

There are challenges and roadblocks to overcome in phytocannabinoid-based therapies. Thus, further research and clinical studies with standardized clinical data and a standardized formulation of phytocannabinoid-containing products are required in order to fulfill the growing demand for plant-based medicines to achieve the optimal therapeutic benefits.

## 8. Future Perspectives and Research Recommendations

An important advance in phytocannabinoid research requires exploration of structure–activity relationships (SARs) to determine how phytocannabinoid molecular modifications affect the selective binding and affinity for SGLT2, as well as other renal or metabolic targets. Changes in the length of alkyl side chains or substitutions of functional groups may elicit activity, as research indicates moderate SGLT2 interaction with tetrahydrocannabivarin (THCV) and cannabidiol (CBD) [95]. However, there is a gap in defining the essential phytocannabinoid structure that achieves therapeutic efficacy. Ref. [31] shows that THCV improves insulin sensitivity and glucose tolerance. Can further optimizations to its structure improve efficacy without compromising safety? It is imperative that SAR studies lead to reliable phytocannabinoid derivatives for SGLT2 control, with minimized off-target binding.

In silico docking and high-throughput screening of phytocannabinoid derivatives can prioritize leads for preclinical testing. Computational studies have revealed that Cannabis-derived compounds have the potential to bind SGLT2, although direct comparative potency with synthetic inhibitors is lacking [27]. Ref. [55] propose that phytomedicines have a wide therapeutic application for glycemic management, but further experimental validation is needed for this to be confirmed. This requires better integration of in silico results with in vitro and in vivo experiments to demonstrate the efficacy of these therapies for improving glycemic parameters, as well as to select the most promising phytocannabinoids to advance development.

Medicinal chemistry campaigns will be useful for the creation of cannabinoid analog libraries. Based on SAR studies and in silico predictions, the phytocannabinoid structure can be further refined to improve SGLT2 inhibition. It is crucial to profile phytocannabinoid analogs against auxiliary systems, such as CB1 and CB2, as CB1 activation has been shown to accelerate diabetes, whereas CB1 inhibition without unwanted psychiatric effects may improve diabetes-related health outcomes [30]. Selective phytocannabinoid analog activity is essential for therapeutic benefit and minimizing adverse events.

Pharmacodynamic and SAR studies can further explore structure–activity relationships. Renal cell assay studies of glucose uptake, as well as downstream signaling pathways, can improve our understanding of the precise mechanisms of phytocannabinoids that regulate SGLT2 [62,96]. Ref. [55] state that, although phytochemicals are often pleiotropic, the specificity of phytomedicinal application can be improved by identifying active components, the nature of synergistic or antagonistic interactions, and mechanisms of action. These studies will guide targeted therapies for different aspects of T2DM pathology (e.g., glucose reabsorption or metabolic inflammation).

Comparison of phytocannabinoid-based SGLT2 inhibitors with synthetic SGLT2 inhibitors, such as dapagliflozin and empagliflozin, may show how their efficacy compares in glycemic control. Ref. [22] report that these synthetic agents can reduce glucose and glycated hemoglobin through potent SGLT2 inhibition. However, it is important to determine if synthetic SGLT2 inhibitors have any benefit on the wide-spectrum pathology of T2DM, which includes systemic inflammation and oxidative stress. Ref. [79] speculate that the combination of phytochemicals and synthetic SGLT2 inhibitors would have greater benefits. Nevertheless, standardization of experimental models is lacking. Further comparative studies may elucidate the benefits of phytocannabinoid incorporation in treating glycemic imbalance and reducing systemic inflammation.

Head-to-head clinical trials can assess the efficacy of phytocannabinoids and synthetic SGLT2 inhibitors. HbA1c, fasting plasma glucose, and urinary glucose should be primary outcome measures, with further secondary analyses assessing their effect on renal and cardiovascular outcomes. Ref. [31] reports that THCV may improve pancreatic β-cell function, which synthetic SGLT2 inhibitors do not target. These trials will evaluate the comparative benefits of both regimens, with increased sample size to account for heterogeneity between subjects and over varying timescales.

Population stratification can reduce treatment variance in clinical trials. Early evidence from [31] suggests that THCV improves metabolism and has anti-inflammatory benefits. Differences in treatment response between participants must be addressed. Stratification according to genetic, metabolic, and behavioral backgrounds may mitigate the potential for confounding variability in clinical trials. It is also critical to develop therapeutics that are accessible to different ethnic populations, as well as underrepresented populations suffering from diabetes.

Combination of phytocannabinoids and synthetic SGLT2 inhibitors is an innovative strategy for glycemic management and overall health. Ref. [79] suggests that plant-derived remedies would be beneficial to use in combination with synthetic remedies to improve overall health. Future trials may evaluate a combination regimen in treating glycemic imbalance, cardiovascular disease, and CKD progression. Preliminary investigations will determine if synergistic interactions are effective, without inducing detrimental consequences, which must be addressed to develop efficient treatment strategies.

Safety monitoring will be required. The complexity of phytocannabinoid-adverse events must be addressed in a robust safety assessment. Unlike synthetic SGLT2 inhibitors, phytocannabinoids have a wide biological impact and can affect the endocannabinoid system. For example, Ref. [30] propose that the impact of CB1 activation during diabetes progression warrants CB1 inhibition, without incurring psychiatric side effects in vulnerable populations. These safety assessments must be comprehensive, with particular attention to the cardiovascular and renal health risks that require long-term monitoring.

Clinical trials would greatly benefit from expanding beyond the assessment of glycemic indices alone and measuring other validated biomarkers. As reported in [55], phytomedicine reduces systemic inflammation and oxidative stress. This evidence shows that incorporating further assessments in clinical trials can unveil additional health benefits not addressed by synthetic SGLT2 inhibitors, to better appreciate the potential of phytocannabinoid therapies.

Poor bioavailability due to extensive first-pass metabolism requires formulation-based technologies. This will address the major limitation of cannabinoids, such as CBD. Ref. [33] highlight the fact that several formulations that maximize the bioavailability of cannabinoids have been researched extensively. Some examples include nanoemulsion formulations, self-emulsifying drug delivery systems, and oral dosage capsules. While these novel formulations enhance cannabinoid bioavailability to achieve a consistent therapeutic dose, further work is required to scale up formulation parameters that can be used in human subjects.

Standardization of phytocannabinoid extraction, purification, and preparation will reduce therapeutic variability. Ref. [33] explain that different strains and extraction methods produce highly variable products. Standardization would limit the inconsistency in the dosage of phytocannabinoid-based treatments due to biological variability in growth, production, and purification methods. For example, high-performance liquid chromatography (HPLC) can limit batch-to-batch variance in pharmaceutical formulation. Further standardization and harmonization with regulations will be essential.

A full phytochemical profile using mass spectrometry and metabolomics can be assessed to reveal novel phytocannabinoid-related active compounds, synergies, and impurities. Ref. [33] argue that, while cannabis has been consumed for thousands of years, further research is needed on its phytochemical profile and the impact it has on therapeutic outcomes. Identification of these phytocannabinoids can enhance clinical use by maximizing therapeutic efficacy and minimizing off-target events.

Formulation stability must be tested under diverse storage and administration conditions to avoid therapeutic variability. It is critical to assess formulation potency and efficacy under various conditions to ensure it remains safe during its shelf life. Formulation stability must be tested as a major step towards creating pharmaceutical-grade cannabinoid products. Coordination of regulators and formulators to expedite the development of phytocannabinoid therapies is necessary for pharmaceutical-grade product development. Ref. [55] mention the necessity of harmonizing regulatory requirements and addressing the obstacles in approving pharmaceutical-grade phytocannabinoids. Improving the clarity of standards and label claims for these medicines can expedite the pathway for clinical use.

Combined phytocannabinoid and synthetic SGLT2 inhibitor therapies must address the overall pathology of T2DM and other complications. Ref. [79] mention that the benefit of combining synthetic remedies with phytocannabinoids is that, while synthetic medicines improve glycemic control, the incorporation of phytochemicals can prevent or reduce cardiovascular- and renal-related comorbidities. In this way, further development should be targeted at the beneficial effects that phytocannabinoids can offer in T2DM-related complications to assess whether they can serve as a preventative treatment in reducing the mortality rate and/or in slowing down the progression of cardiovascular diseases or kidney diseases. Preclinical analyses are necessary for this combination treatment to fully understand pharmacokinetic interactions and synergic or antagonistic effects. Subsequently, these studies will promote clinical studies focusing on its efficacy and safety.

Novel treatment regimens that combine phytocannabinoids and other conventional therapies (e.g., GLP-1 agonist and DPP-IV inhibitor) can be implemented in comprehensive diabetic management. Ref. [55] conclude that several phytochemical compounds should be incorporated into the treatment regimen of diabetes and its comorbid conditions. These therapies can prevent or alleviate the overall pathology of T2DM and can improve the quality of life for diabetic individuals. For this development, it will be essential to assess the beneficial and detrimental effects of phytocannabinoids on various sub-patient populations, based on T2DM pathology and the co-occurrence of other comorbidities.

The complexity of the phytocannabinoid space and its therapeutic potential must be addressed. An important advance for phytocannabinoid research requires exploration of structure–activity relationships (SARs) to determine how phytocannabinoid molecular modifications affect the selective binding and affinity for SGLT2, as well as other renal or metabolic targets. Changes in the length of alkyl side chains or substitutions of functional groups may elicit activity, as research indicates moderate SGLT2 interaction with THCV and CBD [47]. It is imperative that SAR studies lead to reliable phytocannabinoid derivatives for SGLT2 control, with minimized off-target binding. In silico docking and high-throughput screening of phytocannabinoid derivatives can prioritize leads for preclinical testing. Regarding the optimization of chemical properties and detailed SAR investigations, this phase critically involves rational molecular design aimed at enhancing therapeutic efficacy and safety. Medicinal chemistry campaigns are essential for generating libraries of phytocannabinoid analogs, allowing for a systematic refinement of their structures to optimize SGLT2 inhibition and overall pharmacological profiles.

Implementation of novel formulation techniques and streamlined regulations is required to develop high-quality, pharmaceutical-grade phytocannabinoid therapies. Coordinated efforts among researchers, formulators, and regulatory bodies will accelerate the phytocannabinoid treatment process, improving access and outcomes for those suffering from T2DM.

## 9. Conclusions

In summary, this review has shown that, although synthetic SGLT2 inhibitors effectively promote glucosuria and improve glycemic control in T2DM, their clinical utility is constrained by risks of genitourinary infection and limited metabolic benefits. Phytocannabinoids—most notably THCV, CBD, CBG, and β-caryophyllene—emerge as promising multi-target agents that not only interact with SGLT2 in silico, in vitro, and in vivo but also exert anti-inflammatory, antioxidant, and organ-protective effects via cannabinoid receptors, PPARs, and TRP channels. Early clinical data with THCV indicate improvements in fasting plasma glucose and β-cell function without psychiatric side effects, yet widespread application is hindered by poor oral bioavailability, pharmacokinetic variability, lack of standardized formulations, and regulatory barriers.

To translate these findings into novel diabetes therapies, future work must address several critical areas. Firstly, rigorous structure–activity relationship (SAR) studies are imperative to precisely define how molecular modifications of phytocannabinoids affect their selective binding and affinity for SGLT2, as well as other relevant renal or metabolic targets. Secondly, the development and standardization of advanced delivery systems are crucial to overcoming the significant bioavailability challenges and pharmacokinetic variability inherent to many phytocannabinoids. This includes scaling up nanoemulsion formulations, self-emulsifying drug delivery systems, and oral dosage capsules for human subjects to ensure consistent and predictable therapeutic doses. Next, the conduct of rigorous, large-scale, multi-ethnic clinical trials is essential to comprehensively evaluate metabolic, renal, and cardiovascular endpoints across diverse patient populations. These trials should include head-to-head comparisons with synthetic SGLT2 inhibitors and assess long-term safety, particularly in high-risk populations, monitoring not only glycemic indices but also broader biomarkers of inflammation, oxidative stress, and organ function.

On the other hand, the exploration of phytocannabinoid–synthetic inhibitor combination regimens holds significant promise for a holistic, multi-mechanistic approach to complex metabolic disease management. Such strategies aim to leverage the pleiotropic benefits of phytocannabinoids to address complications that synthetic inhibitors do not target, thereby improving overall patient outcomes and potentially preventing or slowing the progression of comorbidities. Finally, establishing clear and harmonized regulatory frameworks for phytocannabinoid medicines is paramount to facilitate research, ensure quality control, and expedite the pathway for clinical approval and widespread access. This involves standardizing cultivation, extraction, purification, and preparation methods to minimize batch-to-batch variability and ensure product consistency.

This comprehensive exploration, particularly the detailed comparative analysis of phytocannabinoids’ multi-target efficacy and safety profile against the single-target action of synthetic SGLT2 inhibitors, along with a precise roadmap for overcoming current translational barriers, represents the core novelty of this review. By systematically addressing these challenges, phytocannabinoids may complement or even extend the benefits of current SGLT2 inhibitors, offering a holistic, multi-mechanistic approach to complex metabolic disease management.

## Data Availability

No data were produced from this study; all data used are contained in this article and published papers in the references.
